# Streamflow Prediction in Highly Regulated, Transboundary Watersheds Using Multi‐Basin Modeling and Remote Sensing Imagery

**DOI:** 10.1029/2021WR031191

**Published:** 2022-03-24

**Authors:** Tien L. T. Du, Hyongki Lee, Duong D. Bui, L. Phil Graham, Stephen D. Darby, Ilias G. Pechlivanidis, Julian Leyland, Nishan K. Biswas, Gyewoon Choi, Okke Batelaan, Thao T. P. Bui, Son K. Do, Tinh V. Tran, Hoa Thi Nguyen, Euiho Hwang

**Affiliations:** ^1^ Department of Civil and Environmental Engineering University of Houston Houston TX USA; ^2^ Danang Institute for Socio‐Economic Development Da Nang Vietnam; ^3^ National Center for Water Resources Planning and Investigation Ministry of Natural Resources and Environment Hanoi Vietnam; ^4^ Swedish Meteorological and Hydrological Institute Norrköping Sweden; ^5^ School of Geography and Environmental Science University of Southampton Southampton UK; ^6^ Hydrological Sciences Laboratory NASA Goodard Space Flight Center Greenbelt MD USA; ^7^ International Center for Urban Water Hydroinformatics Research & Innovation Incheon Republic of Korea; ^8^ Flinders University Adelaide SA Australia; ^9^ Department of Civil and Environmental Engineering Tokyo Metropolitan University Tokyo Japan; ^10^ Department of Water Resources Hanoi University of Natural Resource and Environment Hanoi Vietnam; ^11^ Faculty of Environment Hanoi University of Mining and Geology Hanoi Vietnam; ^12^ Water Resources Satellite Research Center K‐Water Institute Daejeon Republic of Korea

**Keywords:** multi‐basin model, remote sensing imagery, reservoir operation, Mekong and Vietnam, transboundary, regulated streamflow

## Abstract

Despite the potential of remote sensing for monitoring reservoir operation, few studies have investigated the extent to which reservoir releases can be inferred across different spatial and temporal scales. Through evaluating 21 reservoirs in the highly regulated Greater Mekong region, remote sensing imagery was found to be useful in estimating daily storage volumes for within‐year and over‐year reservoirs (correlation coefficients [CC] ≥ 0.9, normalized root mean squared error [NRMSE] ≤ 31%), but not for run‐of‐river reservoirs (CC < 0.4, 40% ≤ NRMSE ≤ 270%). Given a large gap in the number of reservoirs between global and local databases, the proposed framework can improve representation of existing reservoirs in the global reservoir database and thus human impacts in hydrological models. Adopting an Integrated Reservoir Operation Scheme within a multi‐basin model was found to overcome the limitations of remote sensing and improve streamflow prediction at ungauged cascade reservoir systems where previous modeling approaches were unsuccessful. As a result, daily regulated streamflow was predicted competently across all types of reservoirs (median values of CC = 0.65, NRMSE = 8%, and Kling‐Gupta efficiency [KGE] = 0.55) and downstream hydrological stations (median values of CC = 0.94, NRMSE = 8%, and KGE = 0.81). The findings are valuable for helping to understand the impacts of reservoirs and dams on streamflow and for developing more useful adaptation measures to extreme events in data sparse river basins.

## Key Points

- Satellite images are useful to estimate daily storage volumes for within‐year, over‐year reservoirs, but not for run‐of‐river reservoirs
- The proposed framework can improve representation of local dams in the global reservoir databases and human impacts in hydrological models
- Daily regulated streamflow can be reasonably modeled by an integrated reservoir operation scheme of a multi‐basin model and satellite images

## 1 Introduction

Increasing water demand as a result of growing populations and agricultural intensification, alongside concerns over the adverse impacts of anthropogenic climate change, mean that there is a need to accurately estimate surface water availability to support the sustainable allocation and management of water resources across time, space, and sectors. Nevertheless, particularly in developing countries, sparse in situ monitoring networks can make it challenging to develop reliable solutions, especially in transboundary watersheds, where data access is difficult due to security concerns, technical barriers, and weak water governance (Shiklomanov et al., 2002; Thu & Wehn, 2016). Moreover, more than half of Earth’s rivers are already impounded, and more will be required to meet growing demands for irrigation, water supply, energy, and flood management (Boulange et al., 2021; Grill et al., 2019). As river impoundments change natural hydrological regimes (Boulange et al., 2021; Dang et al., 2020; Zajac et al., 2017), the availability of surface water resources downstream become increasingly reliant on the way in which upstream reservoirs (including, sometimes, in different geopolitical jurisdictions) are operated.

Regulated streamflow predictions have in the past been made by applying calibration functions to simulations of unregulated streamflow (Hoang et al., 2019; Lauri et al., 2012; Masaki et al., 2017), or by seeking to include within hydrological models for direct representations of human‐water interactions (Arheimer et al., 2017; Dang et al., 2020; Shin et al., 2019; Terink et al., 2015; Zhao et al., 2016). These approaches, however, tend to work most effectively within watersheds that have extensive gauging networks or published rule curves. For transboundary watersheds where operational reservoir data are limited, alternative estimation approaches have been developed based on generalized reservoir operation schemes that employ alternatively available data, such as simulated inflow, intended purposes, and reservoir storage volumes (De Vos, 2015; Döll et al., 2003; Hanasaki et al., 2006; Wisser et al., 2010). Unfortunately, Han et al. (2020) found that each of these approaches is only applicable to a particular type of reservoirs, such as the Natural Lake Outflow Scheme for small within‐year reservoirs (Döll et al., 2003) and the non‐irrigation reservoir operation scheme for within‐year reservoirs with significant storage variations (Hanasaki et al., 2006). Using a single, generalized scheme across the different types of reservoirs found within national or sub‐continental scale watersheds thus adversely affects model reliability and the water resource management plans that rely on the modeled outputs (Pechlivanidis & Arheimer, 2015).

Recently, satellite imaging sensors and altimetry have been successfully used to support reservoir operation monitoring (e.g., by generating time series of reservoir storage volumes) and the identification of reservoir operation pattern. Although the previous studies (Table 1) indicate that remote sensing imagery has been used to constrain large‐scale changes in reservoir storage volumes through time, there has been a lack of studies investigating the extent to which reservoir releases can be inferred across different spatial scales and at varying temporal resolutions. For example, flow can be regulated at daily time scales for run‐of‐river (ROR) dams, or seasonally within a year (within‐year) and over multiple years (over‐year) for large dams (Vogel et al., 1999, 2007). Small storage reservoirs, mostly small hydropower plants (SHP) operating as ROR schemes, do not have operation rules but respond to sub‐daily flow variations to address high energy demands during peak hours (Almeida et al., 2020; Ashraf et al., 2018; McManamay et al., 2016). Large storage reservoirs, depending on annual variations of inflow (*C*_v_), could be designed to regulate flows within or over a year for one or more functions (e.g., irrigation, hydroelectricity, water supply, and flood control). These larger dams tend to be operated according to operation rules, schedules, and guides approved by water authorities (Vogel et al., 1999). Despite the advantages of the wide spatial coverage of remote sensing imagery, the low temporal resolutions of many sensors (e.g., 16 days for Landsat and 12 days for Sentinel‐1; Sheffield et al., 2018) can hinder its potential to be used to monitor the daily operation of reservoirs, a limitation to which small reservoirs are particularly sensitive.

**Table 1:** Studies Performing Statistical Evaluation of Satellite‐Based Reservoir Operation

Study	Satellite observations	Evaluated region	Evaluated variables	Methods	Main findings
Gao et al., 2012	Satellite altimetry and Moderate Resolution Imaging Spectroradiometer (MODIS)	5 large‐scale reservoirs in the United States from 1992–2010 at monthly scale	Surface areas, water levels, storage volumes	(1) Delineated mask with the percentiles for the water class was used to generate reservoir boundary; (2) Storage volumes were obtained using trapezoidal approximation from overlapping dates of satellite altimetry and MODIS.	(1) The storage estimates were highly correlated with observations (≥0.92), with values for the normalized root mean square error (NRMSE) ranging from up to 15%; (2) Nevertheless, information of validated reservoirs' reservoir storage behaviors (i.e., storage, annual inflows) was not provided and satellite altimetry does not cover all lakes and reservoirs on Earth.
Bonnema & Hossain, 2017	Satellite altimetry, Landsat and Shuttle Radar Topography Mission (SRTM) Digital Elevation Model (DEM)	3 within‐year reservoirs in Thailand, Vietnam and United States at monthly scale	Water levels	Water levels were obtained from satellite altimetry or using trapezoidal approximation from Landsat‐based surface areas and SRTM‐derived Area‐Elevation curves.	(1) The NRMSE of satellite‐based water levels compared with observed values ranged up to 20%; (2) Nevertheless, no evaluation was performed for run‐of‐river and over‐year reservoirs and satellite altimetry does not cover all lakes and reservoirs on Earth.
Zhao & Gao, 2018	Landsat	9 reservoirs in the United States, Iraq, Mozambique, Brazil, China, Australia, and India from 1984 to 2014 at monthly scale	Surface areas	(1) Buffering 500 m outward from GRanD shapefile to generate reservoir boundary; (2) Automatic correction algorithm to enhance water area classification of monthly contaminated water mapping images from the Joint Research Center (JRC) Global Surface Water Mapping Layers v1.2 datasets by Pekel et al. (2016).	(1) An algorithm was proposed to automatically correct contaminated optical image classifications (i.e., clouds, shadows) to generate longer historical record length of monthly surface areas; (2) Nevertheless, information of reservoir storage behaviors was not provided; (3) GRanD database does not include sufficient local lakes and reservoirs. Also, the JRC dataset is only available at monthly scale.
Park et al., 2020	Sentinel–1 Synthetic Aperture Radar (SAR) and SRTM DEM	6 large‐scale reservoirs in China, Brazil, Ethiopia, India, and Venezuela derived from satellite altimetry	Water levels	(1) Density slicing was used to extract reservoir boundary; (2) Water levels were obtained from Sentinel‐1‐based surface areas and SRTM‐derived Area‐Elevation curves.	(1) Estimated water levels agreed well with satellite altimetry‐derived water levels with around 10% of NRMSE; (2) Nevertheless, no evaluation was performed for small‐scale reservoirs.
Weekley & Li, 2021	Landsat and various DEMs	46 water bodies in the United States	Water levels	(1) Canny Edge Detection was performed from ≥85% water occurrence of the JRC datasets to extract reservoir boundary (2) Water levels were obtained from Landsat‐derived surface areas and proportional hypsometry‐derived Area‐Elevation curves.	(1) The lowest hydroflattened surface with proportional hypsometry improved temporal resolution and accuracy of time series of water levels. Among various examined DEMs (ALOS, SRTM, NED30), SRTM DEM was found to be the most accurate model to improve overall accuracy; (2) Nevertheless, information of validated reservoirs' reservoir storage behaviors was not provided; (3) Using optical images requires additional steps to correct contaminated images due to clouds and shadows.
Biswas et al., 2021	Various optical imagery and SRTM DEM	77 reservoirs in India and Bangladesh at monthly scale	Storage changes	(1) Various buffering sizes outward from Global Reservoir and Dam database (GRanD) shapefile were used to generate reservoir boundary; (2) Reservoir storage changes were obtained using trapezoidal approximation from various optical imagery‐based surface areas and SRTM‐derived Area‐Elevation curves.	(1) Estimated storage changes compared with observed values had high correlation coefficient (above 0.7), yet high NRMSE (around 0.5 or more) across all shapes of reservoirs; (2) Nevertheless, information of validated reservoirs' storage behaviors was not provided; (3) GRanD database does not include many local lakes and reservoirs.
This study^a^	Sentinel‐1 SAR and SRTM DEM	21 reservoirs in Vietnam at daily scale. Among them, there are 9 run‐of‐river, 2 over‐year and 10 within‐year reservoirs	Surface areas, water levels, storage volumes and storage changes	(1) Maximum connected water pixels were extracted from the JRC datasets to extract reservoir boundary; (2) Surface areas, water levels, storage volumes and storage changes were obtained using trapezoidal approximation from Sentinel‐1‐based surface areas and SRTM‐derived Area‐Elevation curves.	(1) Maximum connected water pixels were found to generate reservoir Area‐Elevation‐Volumes curves more accurately than using GRanD shapefile or various buffering sizes around GRanD shapefile; (2) Remote sensing imagery was found to be useful in estimating daily storage volumes for within‐year and over‐year reservoirs (correlation coefficients (CC) ≥0.9, NRMSE ≤ 31%), but not for run‐of‐river reservoirs (CC < 0.4, 40% ≤ NRMSE ≤ 270%); (3) This approach can provide reservoir bathymetry for any lakes and reservoirs on Earth and daily reservoir operation dynamics for within‐year and over‐year reservoirs.

^a^ The present study has been added for completeness.

Meanwhile, in spite of recent attempts to create global georeferenced reservoir and lake databases (Biswas et al., 2021; Mulligan et al., 2020; Wang et al., 2021), the available global databases often neglect regional/local dams. Most reservoir studies using satellite observations (Biswas et al., 2021; Li et al., 2020; Zhao & Gao, 2018) rely on the geo‐referenced locations and boundary extents of the most well‐known Global Reservoir and Dam database (GRanD; Lehner et al., 2011). However, there are a large number of smaller reservoirs that are undocumented in GRanD (its latest version provides 7,320 dams) and recently Georeferenced global Dam and Reservoir dataset or GeoDAR (Wang et al., 2021; with about 20,000 dams). For instance, although the number of regulated lakes and reservoirs reported for Sweden and Vietnam was 1 and 40 in GRanD, respectively, their national databases show the true numbers were 125 in Sweden (Arheimer et al., 2017) and 7,000 in Vietnam (MARD, 2015; MONRE, 2020; World Bank, 2015). Additionally, a large number of new reservoirs and dams are continuously being planned and constructed (Bussi et al., 2021; Hecht et al., 2019). Therefore, a framework is needed to generate reservoir operation data for almost any lakes or reservoirs on Earth without relying on the global georeferenced reservoir databases.

As representation of human impacts in hydrological models is still unresolved due to limited understanding of regulation schemes and insufficient reservoir data (Nazemi & Wheater, 2015; Pechlivanidis & Arheimer, 2015), the usefulness of hydrological models and services operating at the large scale is challenged. Accordingly, attempts have been made to estimate daily regulated streamflow in ungauged watersheds through the use of a hybrid mass balance (MB) approach that combines reservoir inflows derived from hydrological modeling with reservoir storage changes estimated from satellite observations (Table 2). However, due to the low temporal resolution of satellite observations, reservoir storage changes at daily temporal resolution (the typical resolution of hydrological models) can only be estimated through temporal interpolation or by averaging across multi‐year operations. The most recent study by Han et al. (2020) (Table 2), nevertheless, shows that the MB approach did not provide reasonable skills for estimating reservoir outflows at cascade reservoirs. An alternative approach for modeling reservoir outflows that is well‐established and widely used is the one that also includes information about zoned water levels (i.e., minimum water levels, spillway water levels) to simulate flow releases according to rule curves and spillway flows when exceeding the maximum water levels (Arheimer et al., 2017; Dang et al., 2020; Klipsch & Hurst, 2007; Zhao et al., 2016). This scheme is termed the Integrated Reservoir Operation Scheme (IROS) in this study. Conventionally, IROS requires observed reservoir outflows to estimate the reservoir parameters. However, it is expected that the averaged reservoir outflows necessary to implement IROS can be obtained from remote sensing imagery and the MB approach.

**Table 2:** Studies Performing Statistical Evaluation of Reservoir Outflows at Ungauged Reservoirs

Study	Satellite observations	Evaluated region	Evaluated variables	Methods	Main findings
Bonnema et al., 2016	Satellite altimetry and SRTM DEM	1 reservoir in Bangladesh and 1 reservoir in United States at annual scale	Reservoir outflows	Mass balance (MB) approach combining hydrological model‐based inflows and satellite‐based reservoir storage changes.	(1) MB method was used to estimate the annual outflow of both reservoirs with reasonable skill (NRMSE of 23.4% and NSE below 0.3); (2) Information of validated reservoirs’ storage behaviors was not provided.
Han et al., 2020	Satellite altimetry	6 reservoirs at daily scale. Among them, there are 2 within‐year reservoirs in China, 1 within‐year reservoir in Thailand, 1 within‐year in the United States and 2 run‐of‐river reservoirs in China.	Reservoir outflows and regulated streamflows	MB approach combining hydrological model‐based inflows and satellite‐based reservoir storage changes.	(1) MB approach was found to estimate daily outflows from individual reservoirs with higher skill (CC > 0.6, NRMSE < 0.2) than other existing generalized operation schemes, but at the cost of higher errors (CC < 0.4, NRMSE > 0.2) when individual reservoirs are combined into a cascade across a watershed; (2) Nevertheless, satellite altimetry does not cover all lakes and reservoirs on Earth.
This study^a^	Sentinel‐1 SAR and SRTM DEM	21 reservoirs in Vietnam and 5 hydrological stations downstream of cascade systems in Vietnam, Laos, Cambodia and Thailand at daily scale. Among them, there are 9 run‐of‐river, 2 over‐year and 10 within‐year reservoirs	Reservoir outflows and regulated streamflows	For within‐year and over‐year reservoirs, Integrated Reservoir Operation Schemes (IROS) parameters were calibrated from averaged reservoir outflows obtained from remote sensing imagery and the MB approach. For run‐of‐river reservoirs, predefined IROS parameters (Section 3.3) were used due to the above findings about capacities of remote sensing imagery (Table 1).	(1) Using IROS approach, daily regulated streamflow was predicted competently across all types of reservoirs, including cascade reservoirs (median values of CC = 0.65, NRMSE = 8%, Kling‐Gupta efficiency [KGE] = 0.55) and downstream hydrological stations (median values of CC = 0.94, NRMSE = 8%, KGE = 0.81); (2) As hydrological models operating at the large scale are still challenged by limited understanding of regulation schemes, the study can guide hydrological model setup over large river systems whose hydrological response is strongly subject to reservoir regulation.

^a^The present study has been added for the sake of completeness.

The aim of this study is to predict daily regulated streamflow within highly regulated, transboundary watersheds using a hydrological model with IROS and remote sensing imagery. In particular, it aims to address the following scientific questions: (a) Can remote sensing imagery support monitoring of reservoir operation patterns across different storage reservoir behaviors? (b) Can IROS, using publicly available data and remote sensing imagery, predict regulated streamflow across individual reservoirs with different behaviors and for cascade reservoirs? The study goes beyond state‐of‐the‐art catchment‐scale practices to improve hydrological model performance in data sparse regions, by focusing on the use of remote sensing to guide hydrological model setup over large river systems, with a particular focus here on catchments whose hydrological responses are strongly subject to reservoir regulation.

## 2 Study Area and Data

**Figure 1:**
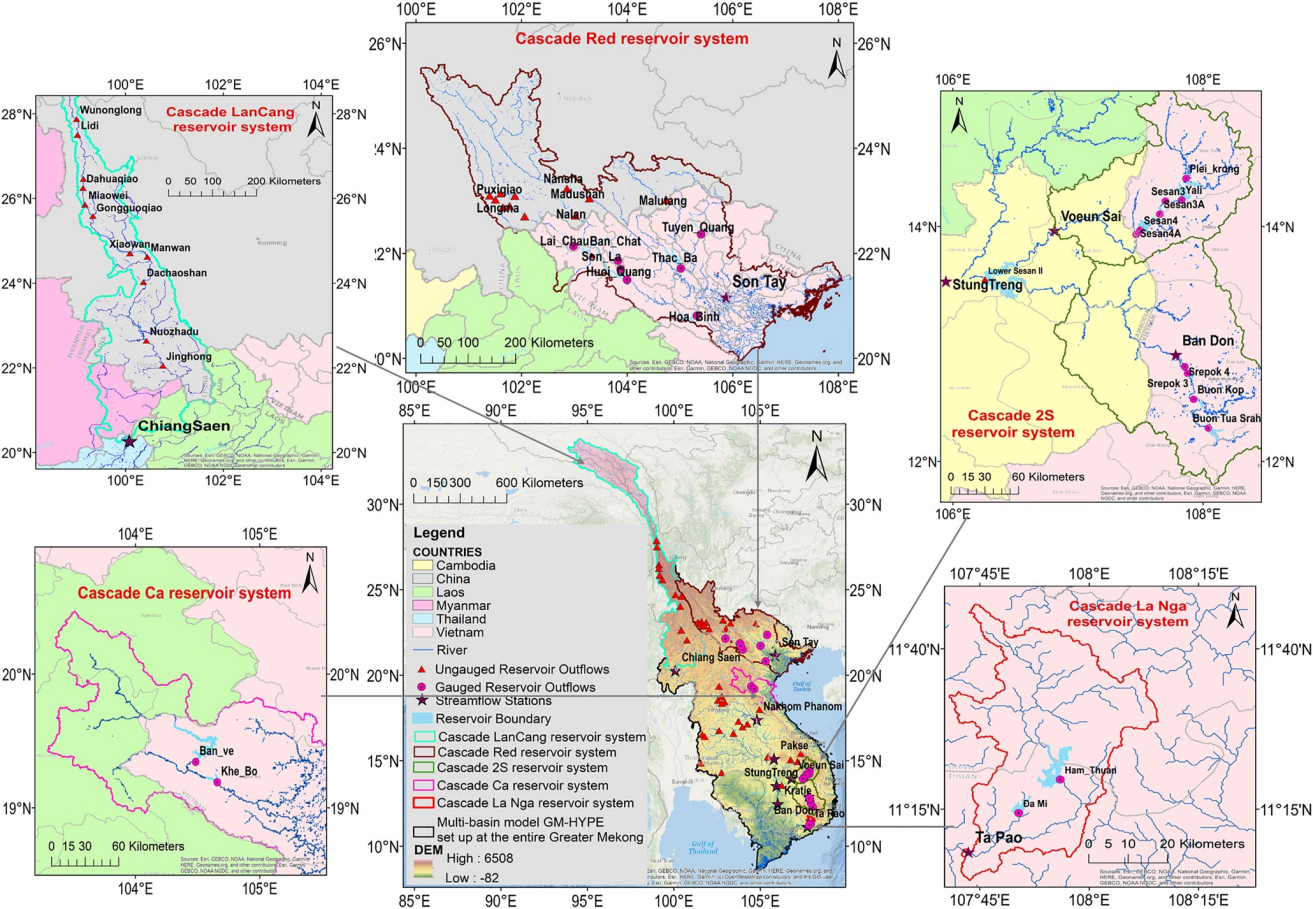
Locations of tested reservoirs and streamflow stations in the Greater Mekong region, where the GM‐HYPE v1.3 and v1.4 were set up.

The Greater Mekong (GM) region (Figure 1), which encompasses six international river basins flowing through China, Myanmar, Laos, Cambodia, Thailand, Vietnam, and seven domestic river basins in Vietnam, has seen a proliferation of hydropower dams (Kittikhoun & Staubli, 2018; Pham, 2015). All 13 river basins in the GM region are transboundary, either between neighboring provinces in one country or between neighboring countries. We focus here on the reservoirs of this region because this study locale offers: (a) diversity of storage reservoir behaviors and properties, (b) diversity of climate zones, and (c) availability of observed data to validate the results. Accordingly, a total of 64 reservoirs and 9 gauging streamflow stations were tested in this study (Figure 1). Among them, 21 reservoirs in Vietnamese territory have observed reservoir data (e.g., AEV, time series of reservoir operation) available to validate the satellite imagery‐derived reservoir operation estimates (see Table S1 in Supporting Information S1). These 21 reservoirs are located in the international Red (7 reservoirs), international Ca (2 reservoirs), international Mekong—Sesan tributary (6 reservoirs), international Mekong—Srepok tributary (4 reservoirs), and the international Dong Nai—La Nga tributary (2 reservoirs) river basins. The remaining 43 reservoirs, all located in neighboring countries, do not have observed reservoir operation data available due to highly restricted data accessibility. These 43 reservoirs were reserved for further analysis to validate the IROS approach using MB‐derived outflow, should it prove to be suitably robust. Additional streamflow observations at nine gauging stations (five of them are international gauging stations) within 20–300 km downstream of cascade reservoirs on both the national and transboundary rivers, were used to validate the simulated regulated streamflow downstream of cascade reservoirs. Descriptions of the reservoir properties and stations along their sources are provided in Table S1 in Supporting Information S1.

Reservoir behaviors are here represented using the reservoir storage ratio (SR). The SR is calculated as *S*/*µ*, where S is reservoir storage volume at capacity and μ is the average annual reservoir inflows that can be simulated using GM‐HYPE v1.4 (for details see Section 3.2). The SR represents the degree of regulation (DOR) of reservoirs (*S*/*µ* × 100), which captures the combined influence of regulation capacity, operation modes, and impacts of individual reservoirs on downstream hydrologic conditions (Grill et al., 2019; Lehner et al., 2011; McManamay et al., 2016; Vogel et al., 1999, 2007). Among 21 reservoirs with observed data, 9 reservoirs are operated as ROR with a mandatory fixed daily minimum environmental flow (Table S1 in Supporting Information S1). All ROR reservoirs have SR values <0.1 (Table S1 in Supporting Information S1). The rest are required to follow rule curves and release a fixed amount of minimum daily flow. The 21 reservoirs operate either for hydropower or multi‐purposes that include hydropower generation.

In addition to the reservoir data, a range of other datasets were used in this study (Table 3). Reservoir inflows were obtained from our updated Greater Mekong—Hydrological Prediction for Environment multi‐basin model (GM‐HYPE v1.3) that has been calibrated and validated using unregulated historical streamflows across 13 main river basins (Du et al., 2020). A detailed description of the data input and model setup for GM‐HYPE v1.3 can be found in Du et al. (2020). GM‐HYPE v1.4 (the version used in this study) is an updated model with the latest reservoir properties and precipitation data. For precipitation, since the Multi‐Source Weighted Ensemble Precipitation v1 (MSWEP v1; Beck et al., 2017), which was used in GM‐HYPE v1.3, is not operational anymore, five operational precipitation products (Table 3) were evaluated using data from 536 rain gauges across the GM region covering the period 2001 to 2017. For remote sensing imagery retrieval and processing, we used Google Earth Engine (GEE; Gorelick et al., 2017). The observed reservoir outflow data for validation were only available from 2016 to 2018 as Ministry of Natural Resources and Environment (MONRE) started to require dam owners to set up an automatic outflow monitoring system only since late 2014 (MONRE, 2017; Vietnamese Government, 2013). Accordingly, Landsat 8 images from January 2016 until December 2018 were used. It is known that Landsat‐8, with improved radiometric resolution and geolocation accuracy, has better capabilities than previous Landsat missions for water mapping (Roy et al., 2014). In addition, geometrically terrain‐corrected Sentinel‐1 SAR Ground Range Detected (GRD) imagery available in GEE was used in the same period (Google Developers, 2020). To derive the AEV relationship, the SRTM DEM (Farr et al., 2007) was used. It was demonstrated that the SRTM DEM provided reasonable hydro‐flattened surfaces for tracking lake surface elevations with mean absolute error of 2.49 m for 46 lakes and reservoirs in the United States (Weekley & Li, 2021).

**Table 3:** Data Sets Used in This Study

Purpose	Variable	Data products	Time span	Spatial resolution	Temporal resolution	References
Hydrological model	Precipitation	Global Precipitation Measurement Integrated Multi‐SatellitE Retrievals for GPM (GPM‐IMERG) v6	2001–2018	0.1°	30 min	Huffman et al., 2019
		the Global Satellite Mapping of Precipitation (GSMaP)	2001–2018	0.1°	Hourly	Kubota et al., 2007
		the Climate Hazards Group Infrared Precipitation with Station Data (CHIRPS)	2001–2018	0.05°	Daily	Funk et al., 2015
		Multi‐Source Weighted Ensemble Precipitation v2 (MSWEP v2)	2001–2018	0.1°	Three‐hourly	Beck et al., 2019
		European Centre for Medium‐Range Weather Forecasts (ECMWF) fifth‐generation (ERA5)	2001–2018	0.25°	hourly	C3S, 2017
Reservoir operation estimation	Water surface areas	Landsat 8 Collection‐1 Tier‐1 Top‐of‐Atmosphere	2016–2018	30 m	16 days	Roy et al., 2014
		Sentinel‐1 SAR Ground Range Detected (GRD) imagery	2016–2018	10 m	12 days	Google Developers, 2020
		Joint Research Centre (JRC) Global Surface Water Mapping Layers v1.3	1984–2021	30 m	Monthly	Pekel et al., 2016
	Area‐Elevation‐Volumes Curves	Shuttle Radar Topography Mission (SRTM) Digital Elevation Models (DEMs)	2002	30 m		Farr et al., 2007

*Note.* Detailed description of other data (e.g., temperature, topography) can be found in Du et al. (2020).

## 3 Methodology

To address the research questions stated in Section 1, we follow three main steps in the methodology: (a) developing a framework to generate reservoir operation data using remote sensing imagery; (b) upgrading the multi‐basin hydrological model from GM_HYPE v1.3 to GM_HYPE v1.4; (c) estimating reservoir outflows and regulated streamflow using a combination of remote sensing imagery and IROS within the GM_HYPE v1.4.

### 3.1 Reservoir Operation Data From Remote Sensing Imagery

**Figure 2:**
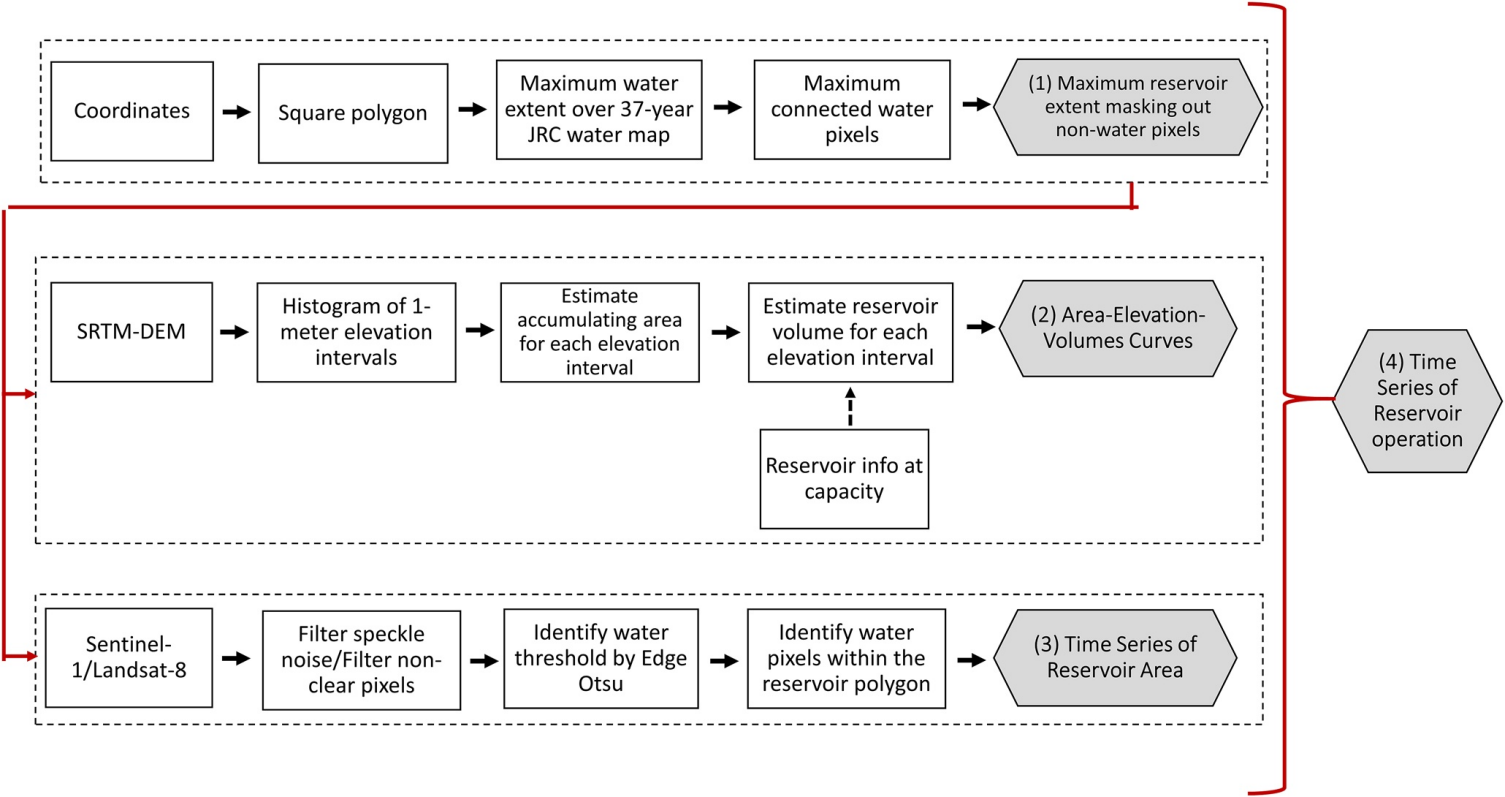
The remote sensing imagery based reservoir operation framework for generating reservoir operation data.

The remote sensing imagery framework for deriving reservoir operation is illustrated in Figure 2. There are four key sections in the framework. The first section is to extract reservoir boundaries using the Joint Research Centre (JRC) Global Surface Water Mapping Layers v1.3, of which errors of commission and omission are <5% (Pekel et al., 2016). The second section is to derive the AEV relationships using the SRTM DEM, following well‐established methods from previous studies (Biswas et al., 2021; Bonnema et al., 2016; Gao et al., 2012; Weekley & Li, 2021). The third section is to generate time series of reservoir surface areas using the Edge Otsu threshold method (Donchyts et al., 2016) for Landsat‐8 and Sentinel‐1 imagery. The final section is to generate time series of interpolated and averaged reservoir operation rules (i.e., elevations, storage volumes, and storage changes). Details for each section are now provided below.

#### 3.1.1 Reservoir Boundary Identification

Defining a reservoir boundary accurately is a critical step in obtaining robust AEV relationships and thus accurate estimates of reservoir extents. Previous studies have mostly relied on the global geo‐referenced reservoirs and dams database from GRanD (Lehner et al., 2011). For this study, first of all, from a center coordinate of a target reservoir, a square polygon covering the reservoir visibly identified from a GEE base map was drawn. Inside this square polygon, a 30‐m maximum water occurrence was identified if water was detected for each pixel from the 37‐yr JRC water classification covering the period 1984 until 2020 (Pekel et al., 2016). Maximum connected water pixels that intersect with the center coordinate of the target reservoir were extracted to define the maximum extent of the reservoir. Since reservoir boundaries are subject to seasonal fluctuations, maximum reservoir extent masking out the non‐water areas throughout the multi‐decadal history of water classification can help identify the peak‐season state of reservoir operation.

#### 3.1.2 Reservoir Area‐Elevation‐Volume Relationship

From the identified maximum reservoir boundary, the relationship between reservoir elevation and reservoir area was determined by classifying the SRTM elevation data into 1 m intervals and estimating the total surface area of each interval inside the identified reservoir polygon. The steepest slope of the reservoir elevation‐area (E‐A) was used to filter out possible SRTM errors (e.g., baseline roll errors, phase errors, beam differential errors, and timing and position errors; Rodríguez et al., 2006) and identify elevations corresponding to reservoir surface areas (Biswas et al., 2021). To estimate the E‐A relationship below the hydro‐flattened water levels from the SRTM DEM (acquired in February of 2000), a power law function was fitted to the lower E‐A relationship for each reservoir. Then, with reservoir area (*A*) and elevation (*h*) from the E‐A curve, the corresponding reservoir volumes (*V*) at each elevation interval (*n*) were computed using a trapezoidal approximation (Equation 1a) with *V*_c_, *A*_c_, and *h*_c_ representing storage, area, and water elevation at capacity, respectively. Otherwise, at lower elevation bands, aggregated reservoir storages for each elevation band were estimated using (Equation 1b) where i=1 is the lowest reservoir elevation obtained from the E‐A relationship, and *i*th is the number of bands from the lowest elevation to the target elevation band *n*th. The values at capacity are published reservoir properties in each country. In this study, for the Vietnamese reservoirs, the reservoir values at capacity were retrieved from public rule curves of multi‐reservoirs in each river basin (Vietnamese Government, 2018). For reservoirs outside Vietnam, the values were taken first from GRanD database and the Mekong Dam Observatory that do not have the same reservoirs reported in the GRanD database (WLE Greater Mekong, n.d.).

(1) $$V_{n}=\;\left\{\begin{array}{ll} V_{c}-\frac{\left(A_{c}+A\right)\left(h_{c}-h\right)}{2} & \;(a)\;if\;V_{c}>\frac{\left(A_{c}+A\right)\left(h_{c}-h\right)}{2}\;\\ \overset{n=1}{\underset{i=1}{\sum }}\frac{\left(A_{i+1}+A_{i}\right)\left(h_{i+1}-h_{i}\right)}{2} & \;(b)\;if\;V_{c}\leq \frac{\left(A_{c}+A\right)\left(h_{c}-h\right)}{2}\; \end{array}\right.$$

The performance of the reservoir elevation‐storage volume (E‐V) and E‐A relationships was then evaluated with the correlation coefficient (CC), and NRMSE between the storage volume or reservoir area associated with each matching elevation as goodness of fit measures between the remotely sensed and observed data (see Table S2 in Supporting Information S1).

Additionally, to evaluate the accuracy of the AEV curves using the maximum reservoir extent identified from Step 1 of the framework (Figure 2), two more approaches to estimate the AEV using GRanD were undertaken. The first approach was to use the original GranD reservoir boundary. As the GranD reservoir boundary might miss the peak‐state season of reservoir operation, Biswas et al. (2021) used different buffering distances of 500 m to 2 km surrounding the reservoir boundary, depending on the total surface area of GranD reservoir polygons. The buffering method adopted in Biswas et al. (2021) was used here as the second approach. In total, 15 out of 21 validated reservoirs that are in the GranD database were used for error analysis for this step.

#### 3.1.3 Reservoir Operation Data

The reservoir surface areas were estimated using either Landsat‐8 or Sentinel‐1 imagery. For Landsat‐8 imagery pixels that are not contaminated (i.e., by cloud, cloud shadow, and snow), the Modified Normalized Water Difference Index (MNDWI; Xu, 2006) was generated to classify water for each pixel (Equation 2), where *X*_*green*_ and *X*_*swir*_ are the reflectance values in the green (Band 3 of Landsat‐8) and short‐wave infrared wavelengths (Band 6 of Landsat‐8), respectively. Here, the Edge Otsu algorithm (Donchyts et al., 2016; Markert et al., 2020), modified from Otsu’s method (Otsu, 1979), was used to identify a unique water threshold across the datasets for each reservoir. Pixels with MNDWI values greater than this threshold were classified as water, while the remaining pixels were assumed as non‐water. The surface area of the target reservoir at the time of the Landsat overpass was then given by the total surface area of detected water pixels within the identified reservoir polygon.

(2) $$MNDWI=\frac{X_{green}-X_{swir}}{X_{green}+X_{swir}}$$

For Sentinel‐1 C‐band SAR GRD imagery, a median filter was first applied to reduce the radar speckle noise (Kim et al., 2020; Lee et al., 2015). The Edge Otsu method as described above was then used to calculate a segmentation threshold across the entire images covering each reservoir during the studied timeline. Once the segmentation threshold was determined, pixels with dB values less than this threshold were classiied as water and otherwise as non‐water. The surface area of the target reservoir was then obtained by summing the detected water pixels within the identified reservoir polygon. Since the revisit periods of Landsat‐8 and Sentinel‐1 are 16 and 12 days, respectively, daily surface area time series were derived by linearly interpolating the generated reservoir area data of the entire time series from January 2016 until December 2018, as done similarly in Han et al. (2020). Averaged values of the 365‐day time series of the reservoir area were then generated by taking the average of the entire interpolated time series for each day of the year.

By combining the reservoir AEV relationship with the time series of reservoir area derived from remote sensing imagery, the corresponding reservoir elevations were identified. This then allowed us to determine the corresponding reservoir storage volumes. Reservoir storage changes (ΔS) for each target day were estimated by subtracting the previous day’s storage volume from the storage volume of the target day. The performance of the daily interpolated historical reservoir areas, elevations, and storage volumes were then evaluated using CC and NRMSE.

### 3.2 Multi‐Basin Hydrological Model GM_HYPE

GM‐HYPE v1.3 was calibrated and validated across 13 main river basins in the GM region using data from 1991 to 2009 (Du et al., 2020) and the GM setup is based on the HYPE hydrological model structure (see Appendix 1 from Lindström et al., 2010). The performance of GM‐HYPE v1.3 was statistically evaluated at 58 gauge stations with Nash‐Sutcliffe Efficiency (NSE), Kling‐Gupta Efficiency (KGE), and CC of 0.66, 0.76, and 0.85 (median values), respectively, during the model calibration period (2002–2009, with 68% of stations having NSE values equal or greater than 0.5) and 0.65, 0.72, and 0.86 (median values), respectively, during the validation period (1991–2001, with 73% of stations having NSE values equal or greater than 0.5; see Table S4 in Supporting Information S1). Any lakes or reservoirs that existed before the year 2009 were already included in GM‐HYPE v1.3. However, GM‐HYPE v1.3 did not model the rule curves of the reservoir operations for these lakes. A total of 29 out of 64 reservoirs that were constructed since 2009 were added by specifying the locations of new outlets and land covers, creating the new version of the model used in this research, GM‐HYPE v1.4. To ensure the correct estimations of evaporation, inflow and outflow, and the locations and properties (i.e., catchment area, surface area at capacity, and diverted or not) of each reservoir were carefully inspected from the national documents for reservoirs in Vietnam and the global reservoir database for the international reservoirs. The lakes and reservoirs are modeled to be located at the outlets of sub‐basins. The outlet lakes and reservoirs receive the outflow from the main river, including all upstream flows and local flows. In GM‐HYPE v1.4 model, the rule curves of the reservoir operation used in the IROS framework are described in Section 3.3.

In principle, HYPE can be forced using precipitation and temperature data at different time steps (e.g., hourly, daily, weekly, monthly, and yearly). Since observed data is at daily resolution, we used this daily time step in this study. To be comparable with the 0.25° (∼28 km) spatial resolution of the temperature reanalysis data from the National Centers for Environmental Prediction Climate Forecast System version 2 (NCEP CFS v2; Saha et al., 2011), five operational precipitation products (described in Section 2) were resampled to this 0.25° grid size. Due to the inherent errors of satellite‐based and reanalysis precipitation products (i.e., sensor errors, retrieval algorithms, and post‐processing procedures; AghaKouchak et al., 2012; Tang et al., 2015), and the fact that hardly any rain gauges in Vietnam are recorded in the global database (Li et al., 2021), it is essential to assess the adequacy of each product in the region. We therefore evaluated five operational products using different statistical metrics (CC, RMSE, and relative error [RE]; Table S2 in Supporting Information S1). It was revealed that each product has different strengths and weaknesses (in terms of correlation, errors, and mean bias) as found in previous studies (Dinh et al., 2020; Dubey et al., 2021; Le et al., 2020). Accordingly, all five products were merged using weighted average spatial correlation for the 536 rain gauges in the region. This approach is similar to the studies by Beck et al. (2017, 2019), which exploited the complementary strengths of each product. Comparing the performance of the new merged product with the 536 rain gauges in the GM, all performance metrics are improved substantially (i.e., CC increased from a median value across the five products of 0.3–0.5; RMSE improved from a median value of 14 to 12 mm/day; RE from median value of −10% to −3%) and the new merged precipitation product was chosen for use in the GM‐HYPE v1.4.

### 3.3 Integrated Reservoir Operation Scheme (IROS)

**Figure 3:**
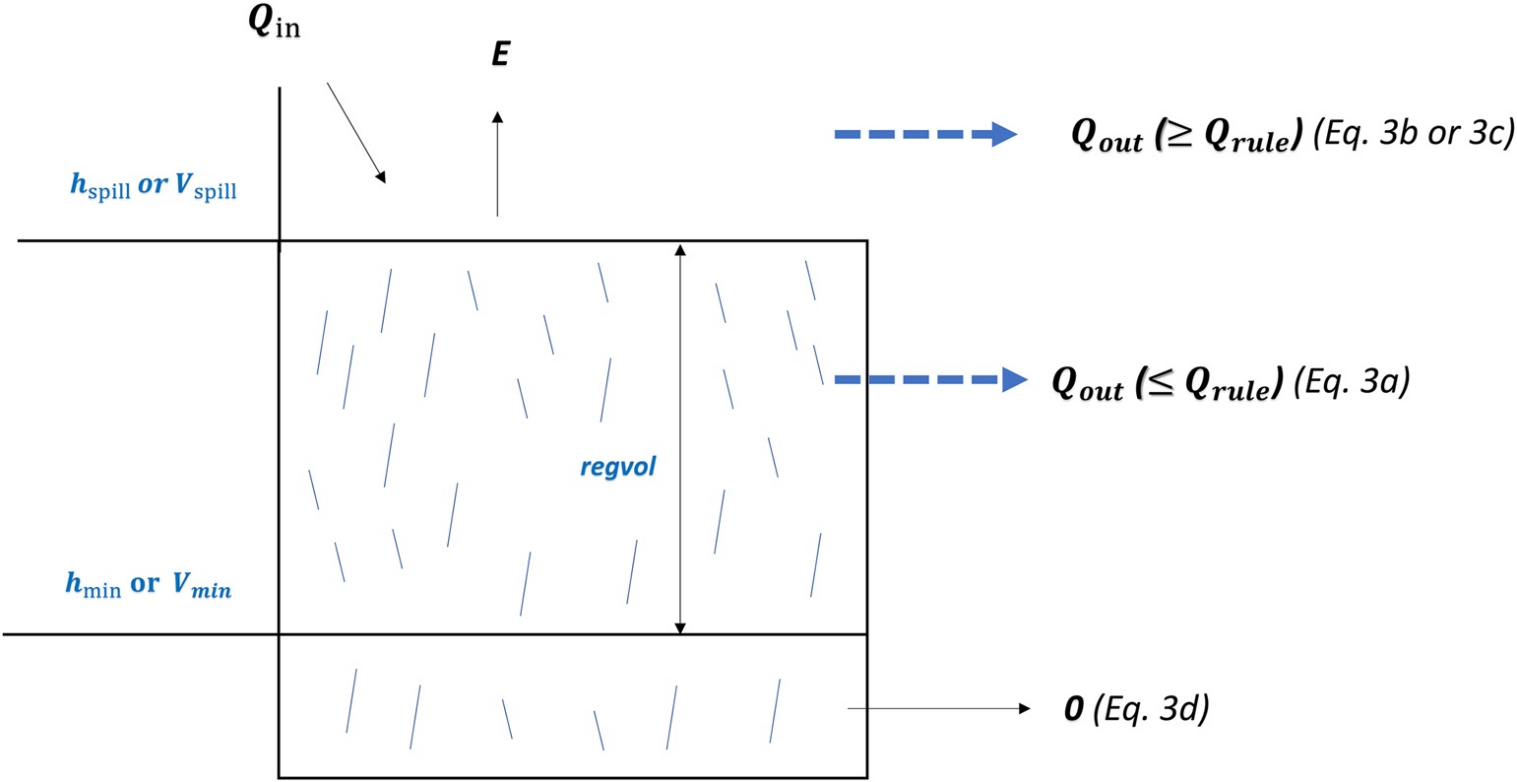
Schematic diagram of Integrated Reservoir Operation Scheme (IROS). *Q*_in_ is reservoir inflow. *E* is evaporation. *Q*_out_ is reservoir outflow calculated from Equations 3 to 6. Regvol is regulation volume of reservoir. *Q*_rule_ is seasonal production flow specified by the rule curve (Equation 5). *h*_min_ or *V*_min_ is minimum water level or volume. *h*_spill_ or *V*_spill_ is spillway water level or volume.

The HYPE model can simulate the rule curves of different types of reservoirs. For hydropower, irrigation, and multi‐purpose reservoirs, the reservoir operation scheme inside the HYPE model is similar to the approach adopted by Klipsch and Hurst (2007) and Zhao et al. (2016). This scheme is termed IROS herein. The IROS simulates the reservoir outflows (*Q*_out_) as follows: (a) at moderate water levels (i.e., between spillway level *h*_spill_ and minimum water levels *h*_min_), outflows follow the seasonal production flows specified by the rule curves (*Q*_rule_). In other words, reservoirs release water (e.g., drier months from December to May) when there are high water demands and store water during periods of lower demands (e.g., during the monsoon season from June to November; Equation 3a); (b) at low water levels, *Q*_out_ is reduced to the amount of water available above minimum water levels (Equation 3a); (c) at spillway levels, outflows are released through the spillways (Equations 3b and 3c; Arheimer et al., 2017; Arheimer & Lindström, 2014); (d) below the minimum water levels, outflows are not released at all (Equation 3d; Figure 3).

The elevation of the spillway, *h*_spill_, and the regulation volume (regvol) can be obtained from the public national reservoir database or can be retrieved using global reservoir data. The minimum water level, *h*_min_, is calculated from regulation volumes (regvol) and lake surface areas at the spillway level (*r*_area_), as shown in Equation 4. Thus, although the HYPE model does not consider the area changing with depth, *h*_min_ and *h*_spill_ represent the minimum reservoir storage volumes (*V*_min_) and the maximum reservoir storage volumes (*V*_max_), respectively. As the observed reservoir water levels in the study region mostly follow sinusoidal variations over the year (e.g., see Figure S1 in Supporting Information S1), the reservoir outflows can be estimated through the seasonal production flows of IROS in this study. The parameters of *Q*_rule_ (*Q*_prod_, *q*_amp_, *q*_pha_) can be estimated individually by simple linear regression using the observed streamflow (Equation 5). In that equation, the variable *Q*_prod_ is the production flow; *q*_amp_ (ranges from 0 to 1) is the amplitude adjusting production flow; *q*_pha_ (ranges from 1 to 365; e.g., 180: peak in October and minimum in April) is the phase shift of the production flow; doy is the day of the year of the target day (Arheimer et al., 2017; Arheimer & Lindström, 2014). In this study, to be applicable for ungauged reservoirs, the average outflows estimated from the MB approach were used to calculate the parameters of *Q*_rule_. No observed reservoir data were used to calibrate the IROS, with observed reservoir data used only to validate the results. The spillway flow is modeled by a rating curve (Equation 6) where h is the water level at the target time step, *h*_spill_ is the spillway water level, and k and p are the rate and exponent of the rating curve parameters, respectively. These parameters are calibrated individually using the observed data. In this study, we set k=0 to simplify the process, and p=2 similar to previous studies (Equation 3b; Du et al., 2020; Lindström, 2016).

(3) $$Q_{out}=\left\{\begin{array}{ll} \min\;\left(Q_{rule},\frac{\left(h-h_{min}\right)\times r_{area}}{t}\right) & \;(a)\;if\;h_{min}\leq h<h_{spill}\;or\;V_{min}\leq V<V_{spill}\\ \text{max}\;\left(Q_{rule},\frac{\left(h-h_{spill}\right)\times r_{area}}{t}\right) & \;(b)\;if\;h\geq h_{spill}\;or\;V\geq V_{spill}\;and\;k=0\;\\ \max\;\left(Q_{rule},Q_{spill}\right) & \;(c)\;if\;h\geq h_{spill}\;or\;V\geq V_{spill}\;and\;k>0\\ 0 & \;(d)\;if\;h<h_{smin}\;or\;V<V_{min}\; \end{array}\right.\;$$

(4) $$h_{min}=h_{spill}-\frac{regvol}{r_{area}}$$

(5) $$Q_{rule}=Q_{prod}\times (1+\times q_{amp}\times \sin(\frac{1\times \pi \times (doy\left(t\right)+\times q_{pha\;}}{365}))$$

(6) $$Q_{spill}=k\times {\left(h-h_{spill}\right)}^{p}$$

The simulated streamflow (i.e., inflow, outflow, streamflow at downstream stations) was evaluated using NSE, NSE for logarithmically transformed flows (NSE_ln_), KGE, CC, relative error (RE), and relative error of standard deviation (RESD). NSE and NSE_ln_ are more suitable for evaluating the goodness of fit for high flows and low flows, respectively (Pushpalatha et al., 2012) whereas KGE and its components (CC, RE, RESD; Gupta et al., 2009; Knoben et al., 2019) are adopted for decomposing and understanding the error components (i.e., timing bias, volumetric bias, and variability bias) of overall flows (see Table S2 in Supporting Information S1).

## 4 Results

### 4.1 Evaluation of Remote Sensing Derived Area‐Elevation‐Volume Curves

**Figure 4:**
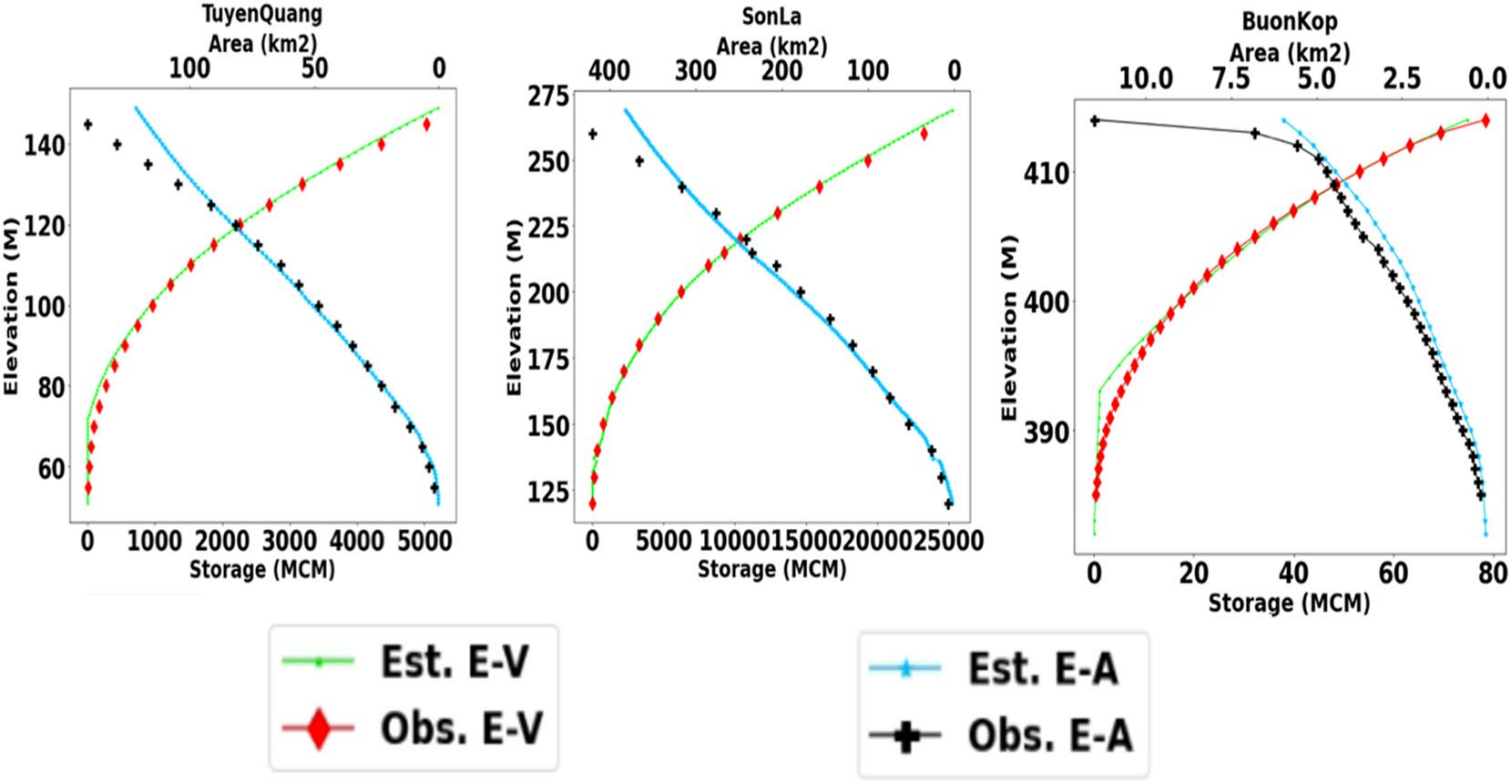
Validation of estimated Area‐Elevation‐Volume (AEV) of three sample reservoirs using the proposed maximum reservoir extent approach (Section 3.1). Tuyen Quang is an over‐year reservoir, Son La is a within‐year reservoir and Buon Kop is an ROR reservoir. “Est.” denotes estimated data. “Obs” denotes observed data.

**Figure 5:**
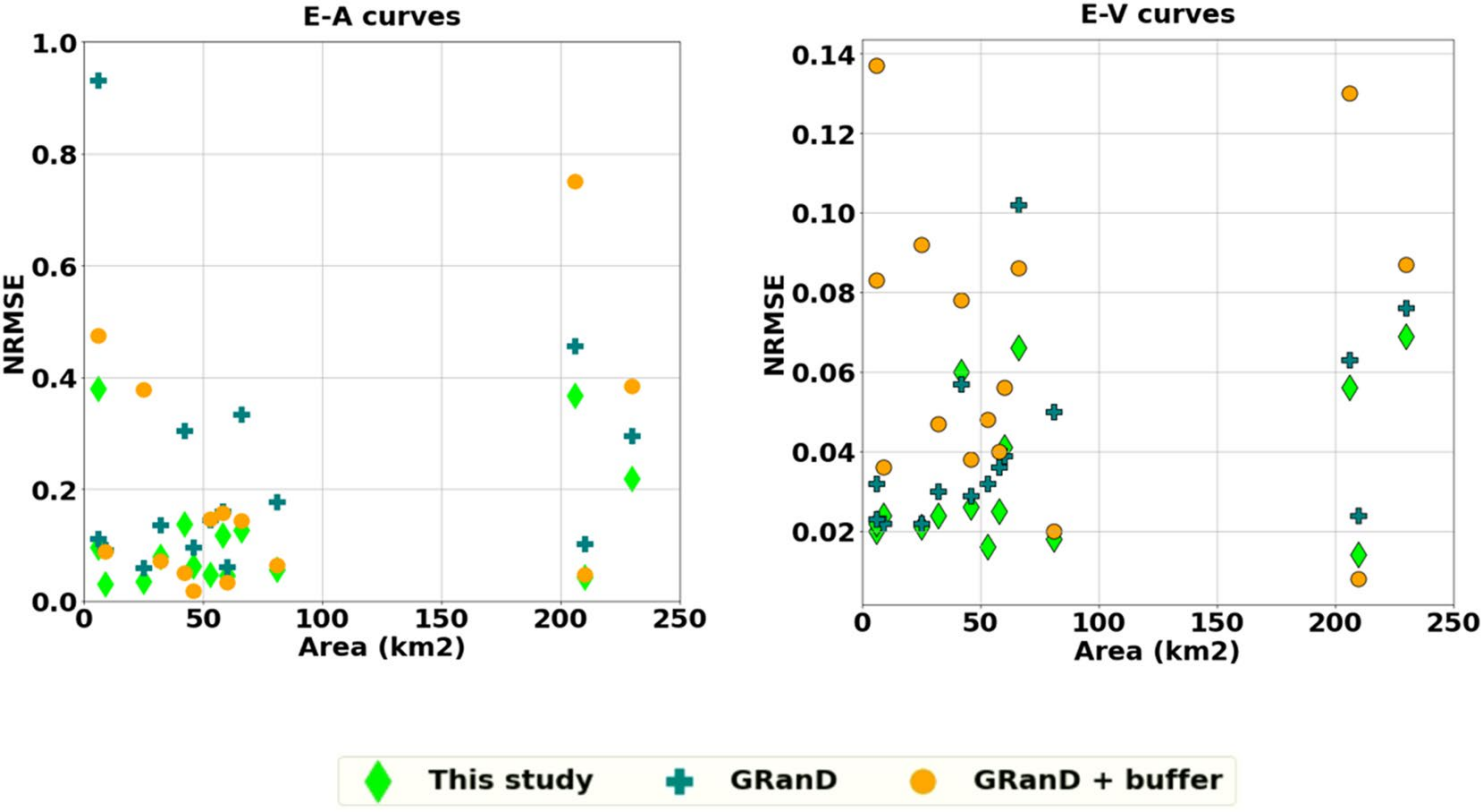
Error analysis of the Elevation‐Area (E‐A) (left) and Elevation‐Volume (E‐V) (right) curves plotted against the areas of reservoirs at spillway elevations using three different approaches (Section 3.1.2) compared with observed data.

A validation of SRTM‐derived AEV curves using observed AEV data was undertaken. Figure 4 shows the comparison of the AEV curves for three reservoirs using the maximum reservoir extent approach proposed in the framework described in Section 3.1. The AEV comparisons for one over‐year reservoir (Tuyen Quang), one within‐year reservoirs (Son La), and one ROR reservoir (Buon Kop) are displayed in Figure 4. The quantitative accuracy of the E‐V and E‐A relationships of 15 reservoirs were assessed using CC and NRMSE as summarized in Table 4. Additionally, Figure 5 plots NRMSE of E‐V and E‐A curves against the surface areas of the reservoirs for all three approaches: (a) the proposed maximum reservoir extent (this study), (b) the reservoir boundary provided by the GRanD database (Lehner et al., 2011), and (c) the GRanD polygons with the buffers proposed by Biswas et al. (2021). It can be seen from Table 4 and Figure 5 that although all three approaches obtained good correlations with observed data (median CC of 0.99), using the GRanD boundaries and the GRanD boundaries with various buffers resulted in higher NRMSE than using maximum reservoir extents. Using the GRanD boundaries alone might result in an under‐estimation of the areas of reservoirs due to the conservative boundary extraction approach as reported by Lehner et al. (2011). Meanwhile, using the GRanD boundaries with various buffers might lead to an over‐estimation of the surface areas since sizes of the buffer zones were determined arbitrarily (Biswas et al., 2021). Overall, the proposed approach appeared to achieve the closest agreement with observed data (E‐V curves: median NRMSE of 2%; E‐A curves: median NRMSE of 8%) compared to other approaches (E‐V curves: median NRMSE of 3%–6%; E‐A curves: median NRMSE of 14%–15%).

**Table 4:** Statistical Evaluation of SRTM‐Derived AEV Relationships

Description	CC	RMSE	NRMSE
E‐A			
This study	0.99 (0.02)	4.4 (km^2^) (15.44)	0.08 (0.13)
GRanD reservoir extent	0.99 (0.04)	10.33 (km^2^) (20.11)	0.15 (0.23)
GRanD reservoir extent + buffer	0.99 (0.12)	8.94 (km^2^) (30.99)	0.14 (0.51)
E‐V			
This study	0.99 (0.00)	41.51 (mcm) (141.02)	0.02 (0.02)
GRanD reservoir extent	0.99 (0.01)	57.22 (mcm) (165.22)	0.03 (0.02)
GRanD reservoir extent + buffer	0.99 (0.02)	89.93 (mcm) (295.28)	0.06 (0.04)

*Note.* The data shown are the median values determined across the 15 reservoirs, with their standard deviations also shown in parentheses. Units are provided for median RMSE. Mcm = million cubic meter.

According to Figure 5, the SRTM‐derived E‐A curves showed higher errors than the E‐V curves due to the quality of the DEM data. Figure 5 suggests that when the reservoir area is below 10 km^2^ and above 200 km^2^, the error (NRMSE) of E‐A estimations increases. Since the SRTM overpassed the region in February of 2000, the lowest reservoir elevations, which often occur in May or June for some reservoirs, might not be fully captured. Small reservoirs with substantial changes within 1 day and large reservoirs with steep bathymetry would thus be more subject to SRTM DEM errors, as similarly found in previous studies (Weekley & Li, 2021; Zhang & Gao, 2020). Nevertheless, as the storage volumes corresponding to each elevation were estimated using the difference from the reservoir storage volume at capacity as calculated from Equation 1, employing the public reservoir data at capacity improved the accuracy of the E‐V estimates.

### 4.2 Evaluation of Remote Sensing Derived Reservoir Operation

**Figure 6:**
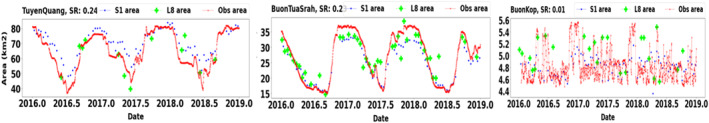
Landsat‐8 (L8 area) and Sentinel‐1 (S1 area) derived time series of reservoir surface area without interpolation compared with observed data (Obs area).

Similarly, the error analyses for the reservoir surface areas, water levels, storage volumes, and storage changes (ΔS) derived from Landsat‐8 and Sentinel‐1 imagery were performed using the observed reservoir data. However, due to many non‐clear pixels in the Landsat‐8 imagery, the median number of available images during the 3‐yr period for 21 reservoirs was only 30, whereas this number for Sentinel‐1 was 94. Figure 6 shows the non‐interpolated time series of Landsat‐8 and Sentinel‐1‐derived reservoir areas compared with the observed areas of one over‐year reservoir (Tuyen Quang), one within‐year reservoir (Buon Tua Srah) and one ROR reservoir (Buon Kop). As noted above, there are far fewer Landsat‐8 observations than Sentinel‐1. Reservoir operations in this study were thus estimated using only the Sentinel‐1 imagery.

**Figure 7:**
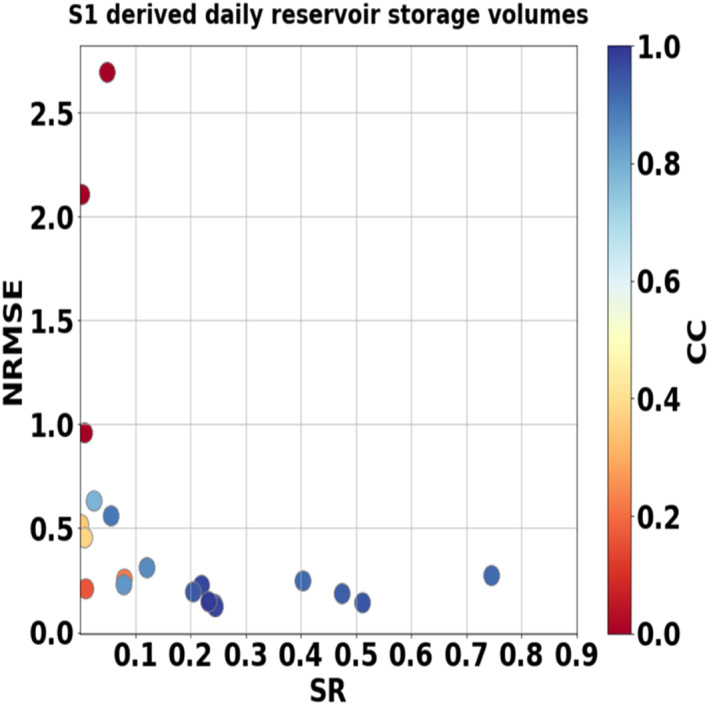
Error analysis of Sentinel‐1‐derived daily storage volumes plotted against Storage Ratio (SR). “S1” denotes Sentinel‐1.

Figure S1 in Supporting Information S1 provides an assessment of the Sentinel‐1 derived reservoir operation data in which time series of the interpolated reservoir surface areas, water levels, storage volumes, and ΔS are compared to observations of one over‐year reservoir (Tuyen Quang), one within‐year reservoir (Son La) and one ROR reservoir (Buon Kop). The error analyses are summarized in Table S3 in Supporting Information S1. It was found that the interpolation process has little impact on the results compared with the non‐interpolation because Sentinel‐1 images were available at least once each month for each reservoir, while within‐year or over‐year reservoirs had distinct seasonal patterns. For ROR reservoirs (Buon Kop) that operated hourly to daily with the “hydropeak,” neither the non‐interpolated nor interpolated time series could capture the right moments of reservoir extent changes due to the low temporal resolution of Sentinel‐1 imagery. This finding is demonstrated by the low CC (<0.3) and high NRMSE (more than 100%) values (Table S3 in Supporting Information S1). NRMSE and CC of the time series of reservoir storage volumes using Sentinel‐1 were plotted against the storage ratio of all reservoirs (Figure 7). Figure 7 shows that there are two distinct patterns for SR values above and below 0.1. In cases when SR was <0.1, CC and NRMSE varied significantly with most of them above 50% of NRMSE and <0.3 of CC. When SR was above 0.1, CC was at least 0.8 and NRMSE was <30%. Similarly, both daily and monthly storage changes had low CC (∼0.4) and high NRMSE (∼30%) for ROR reservoirs, but higher CC (up to 0.87) and lower NRMSE (∼12%) for within‐year and over‐year reservoirs (Table S3 in Supporting Information S1). Between daily and monthly steps, the daily ΔS had slightly higher errors than the monthly one. This was because the daily ΔS which is influenced by several factors (e.g., rule curves, demands, and inflow) could not be captured by Sentinel‐1 imagery with its 12‐day repeat cycle. Overall, it can be concluded that remote sensing imagery, particularly from Sentinel‐1, provides good estimates of within‐year or over‐year reservoirs at both daily and monthly scales, but not for ROR reservoirs.

### 4.3 Model Performance of Reservoir Inflow

**Figure 8:**
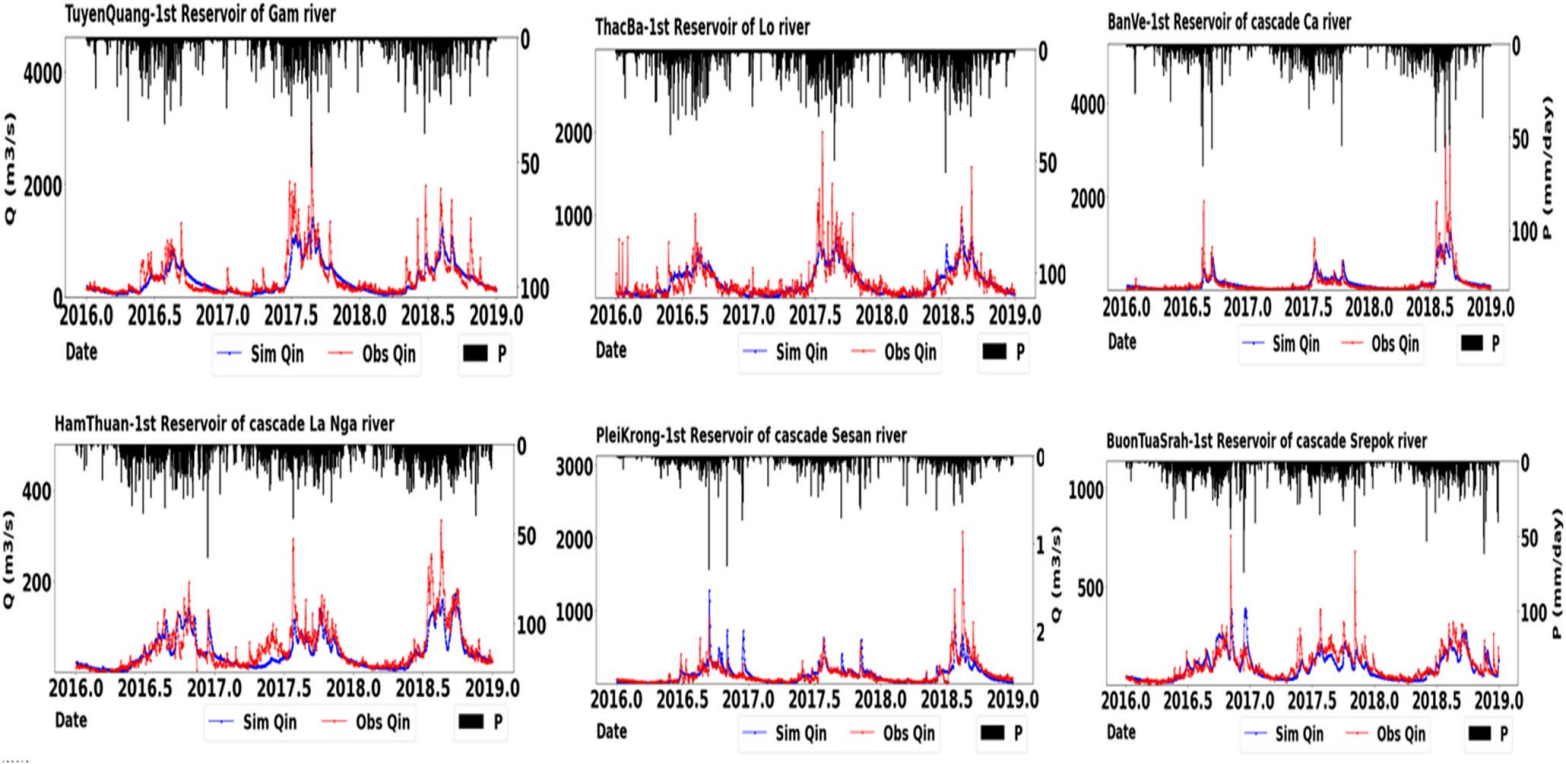
Simulated inflows (*Q*_in_) of the first‐order reservoirs in each cascade system using GM‐HYPE v1.4. “Sim” indicates simulation data from GM‐HYPE v1.4. “P” indicates “precipitation” data.

Figure 8 shows the inflow hydrograph of the first‐order reservoirs within each cascade system. For second and higher‐order cascade reservoirs, the inflow was calculated using the simulated outflow of the upstream reservoirs plus the local flow between two nearby reservoirs. As summarized in Table S4 in Supporting Information S1, GM‐HYPE v1.4 successfully generated both low flows, high flows and overall flows (NSE_ln_ and NSE larger than 0.5 and KGE larger than 0.5). Peak inflows were underestimated at Thac Ba, Ban Ve, and Ham Thuan primarily due to the uncertainty in precipitation data.

### 4.4 Performance of IROS‐Based Reservoir Outflow

**Figure 9:**
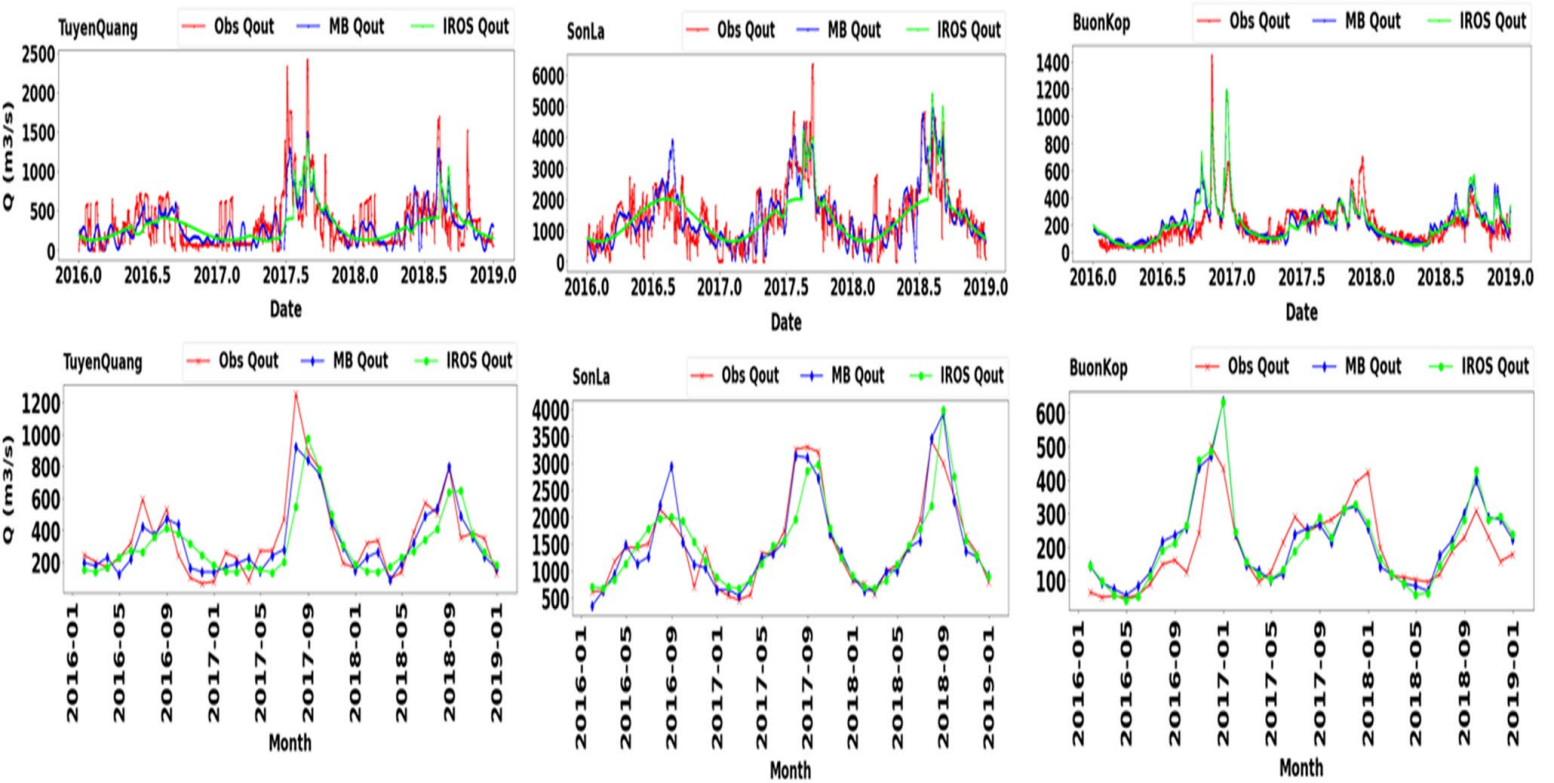
The IROS‐based reservoir outflows (IROS *Q*_out_) compared with the MB‐derived reservoir outflows (MB *Q*_out_) and observed data (Obs *Q*_out_).

The performance of the IROS‐based reservoir outflows is shown in Table 5 and Figure 9 (see Table S5 in Supporting Information S1 and Figure S2 in Supporting Information S1 for IROS‐based ΔS). From Table 5, IROS predicts the outflows relatively well across all types of reservoirs and also reservoir cascades at both daily and monthly steps (KGE, CC of at least 0.5 for both daily and monthly steps, except cascade systems of La Nga river). For the ROR reservoirs, as most of them have small storage volumes, IROS would not substantially change the reservoir releases compared with the inflows, resulting in satisfactory statistical metrics. For cascade systems that have multiple ROR reservoirs (e.g., the Sesan, Srepok cascades, and Lan Cang cascades at Chiang Saen), IROS modeled regulated streamflow satisfactorily (KGE, CC of at least 0.6 for both daily and monthly steps). Compared with the MB approach (see Appendix A and Table S6 in Supporting Information S1), IROS appeared to perform better than the MB approach (Table 5). For both daily and monthly steps using IROS, NSE, KGE, and CC values ranged from 0.6 to 0.9, whereas the metrics for both daily and monthly steps using the MB approach varied from 0.4 to 0.8. For ROR reservoirs, the MB approach might underestimate the spillway flows or overestimate the production flows as it does not consider the limits of reservoir storage capacity. The MB approach can thus introduce larger errors in instances where there are multiple ROR reservoirs in a cascade system.

**Table 5:** Statistical Evaluation of the IROS‐Based Reservoir Outflows and Streamflow at Gauged Reservoirs and Downstream Hydrological Stations

Description	Time step	NSE	NSE_ln_	KGE	CC	RE (%)	RESD (%)
ROR (mostly SR < 0.1)	DD	0.51 (0.22)	0.35 (0.27)	0.75 (0.2)	0.77 (0.15)	1.78 (10.77)	−7.44 (21.2)
	MO	0.6 (0.21)	0.72 (0.25)	0.79 (0.16)	0.83 (0.11)	1.78 (10.92)	−1.72 (21.05)
Within‐year or Over‐year (SR ≥ 0.1)	DD	0.28 (0.22)	0.04 (0.15)	0.51 (0.14)	0.59 (0.16)	−1.63 (8.37)	−34.59 (16.1)
	MO	0.49 (0.23)	0.36 (0.35)	0.65 (0.12)	0.74 (0.13)	−1.56 (8.14)	−21.97 (17.67)
Hoa Binh—Cascade Da	DD	0.38	0.38	0.49	0.62	−4.08	−33.01
	MO	0.55	0.63	0.67	0.75	−5.76	−21.06
Khe Bo—Cascade Ca	DD	0.78	0.62	0.77	0.89	15.31	−13.52
	MO	0.89	0.76	0.82	0.96	15.62	−7.13
Da Mi—CascadeLa Nga	DD	0.10	0.02	0.30	0.45	−15.84	−39.21
	MO	0.25	0.26	0.44	0.61	−15.73	−36.73
Sesan 4A—Cascade Sesan	DD	0.61	0.28	0.63	0.79	7.16	−29.65
	MO	0.72	0.67	0.67	0.87	7.18	−29.26
Srepok 4—Cascade Srepok	DD	0.56	0.38	0.78	0.78	2.25	−1.10
	MO	0.64	0.77	0.82	0.83	2.31	5.38
Vietnamese stations	DD	0.33 (0.26)	0.52 (0.42)	0.64 (0.25)	0.78 (0.09)	22.05 (23.18)	−3.96 (22.67)
	MO	0.36 (0.28)	0.67 (0.38)	0.61 (0.23)	0.84 (0.06)	21.99 (23.21)	3.29 (22.59)
International stations	DD	0.91 (0.14)	0.86 (0.17)	0.83 (0.05)	0.98 (0.08)	8.05 (10.59)	−1.54 (7.45)
	MO	0.93 (0.1)	0.86 (0.12)	0.83 (0.09)	0.98 (0.05)	3.16 (14.89)	0.46 (14.11)

*Note.* Median values are reported with their standard deviations shown in the parentheses. International stations are downstream of ungauged reservoirs. DD: daily; MO: monthly.

### 4.5 Performance of IROS‐Based Regulated Streamflow

**Figure 10:**
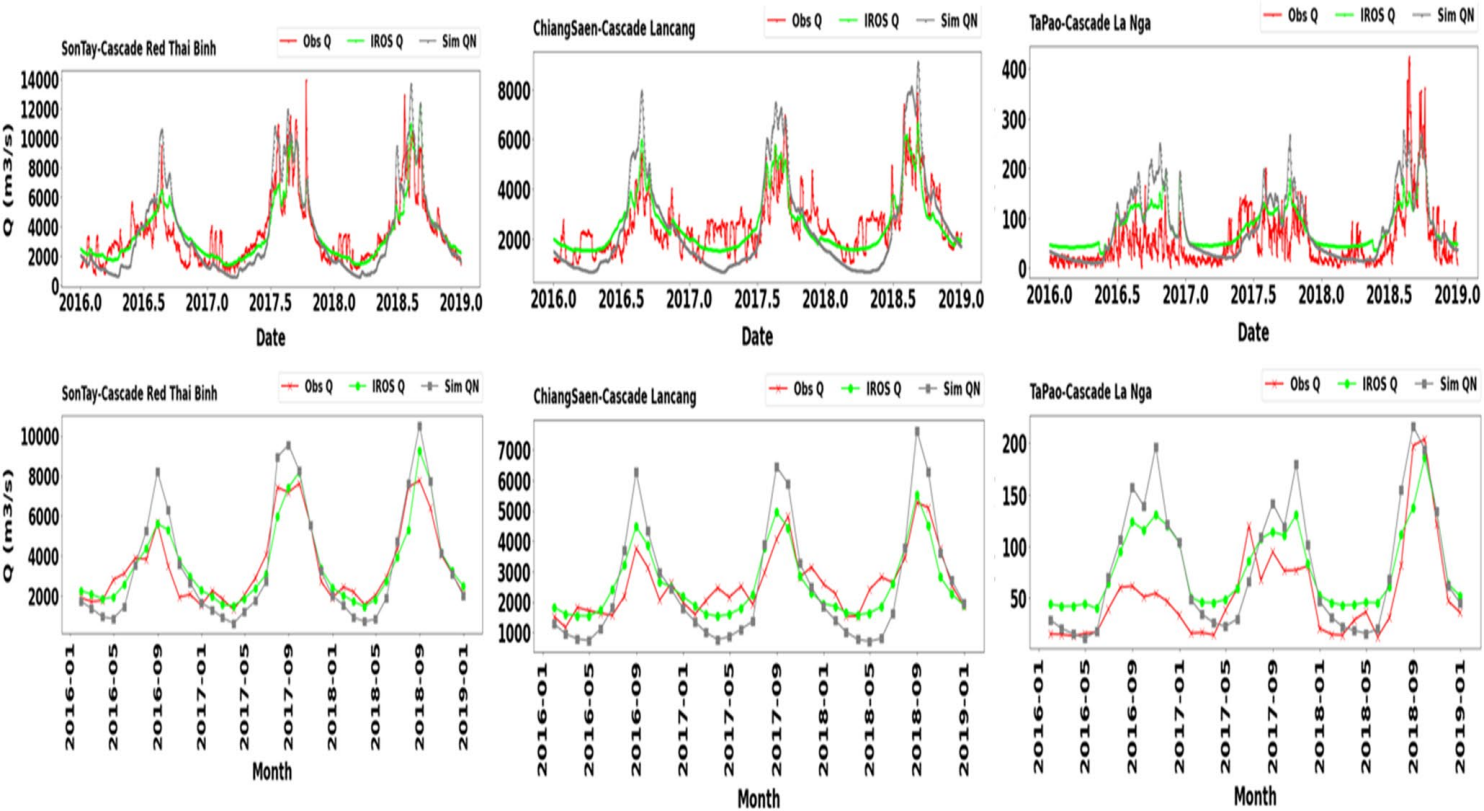
Simulated (Sim) streamflow at the gauged stations compared with the observed (Obs) data and simulated “natural” conditions without reservoirs (Sim QN). The gauged stations are at the downstream of both gauged and ungauged cascade reservoir systems. Son Tay and Ta Pao are at the downstream of the gauged cascade systems whereas Chiang Saen is at the downstream of the ungauged cascade system.

Figure 10 shows the hydrograph of simulated regulated streamflow downstream of cascade reservoir systems, compared with both observed data and “natural” conditions without reservoir operation. The simulated regulated streamflow agreed well with the in situ data. Compared with their “natural” conditions, the regulated streamflows increased during the dry season and decreased during the wet season. At Ta Pao station, which is located downstream of the off‐site hydropower generation reservoirs (i.e., Ham Thuan diverted water to Da Mi in La Nga river), its hydrological impacts were usually more severe than a dam‐site generation because the flows downstream would be substantially reduced. As Ham Thuan reservoir diverted its release to an ROR reservoir (i.e., Da Mi) with hydropeaking, the outflow patterns from the La Nga reservoir cascade became more irregular. Furthermore, in Vietnam, although there were required minimum environmental flows by authorities, exceptions prioritizing energy production were allowed if dam operators requested and got permission from the responsible authorities. The combination of an off‐site hydropower generation and allowable lower minimum flows makes regulated streamflow more irregular for the model to simulate. Therefore, the model performance at Ta Pao stations was worse than that at other stations. Nevertheless, without modeling the reservoir operation, streamflow prediction for highly regulated catchments would be subject to higher errors as visualized in Figure 10.

**Figure 11:**
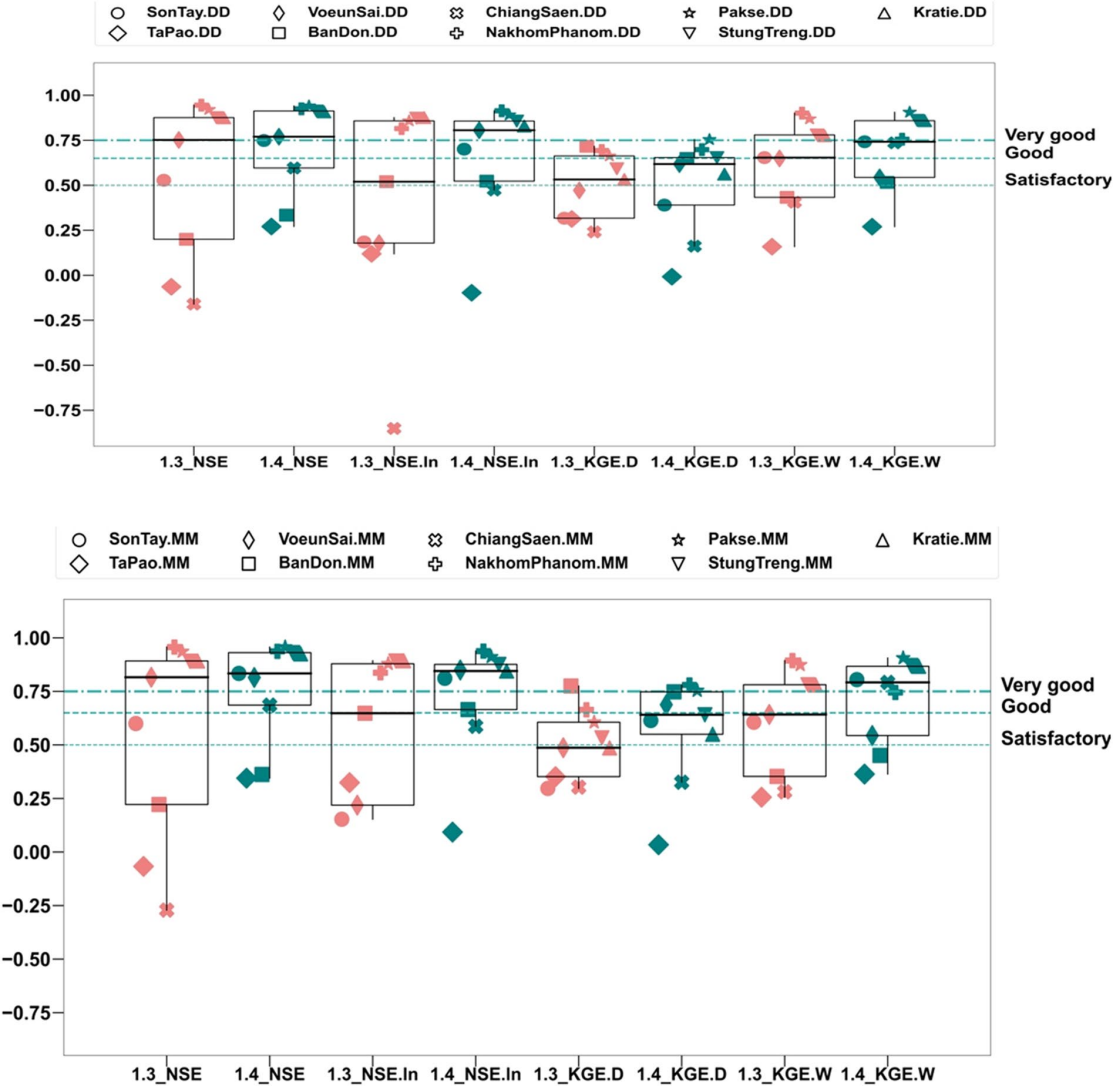
Box plots of the model performance for “natural” GM‐HYPE v1.3 (light red) and “regulated” GM‐HYPE v1.4 (light blue) model versions at both daily (DD) and monthly (MO) steps, in terms of daily high flow dynamics (NSE), daily low flow dynamics (NSE_ln_), overall flow balance in dry season (KGE.D), and in wet season (KGE.W) at hydrological streamflow stations located at the downstream of cascade reservoir systems. Locations of the streamflow stations within the Greater Mekong study region are shown in Figure 1, whereas their detailed description is provided in Table S1 in Supporting Information S1. Model performance rating is based on Moriasi et al. (2007).

Figure 11 shows that the model used herein, GM‐HYPE v1.4, improves the simulation of streamflow of highly regulated stations compared with GM‐HYPE v1.3 that did not include reservoir operation at both daily and monthly time step in terms of high flow dynamics (NSE), low flow dynamics (NSE_ln_), overall flow balance in dry season (KGE_D), and overall flow balance in wet season (KGE_W). The stations are located downstream of cascade reservoirs. Compared to GM_HYPE v1.3, except Ta Pao that consistently had unsatisfactory performance in all selected metrics (below 0.5; reasons explained above), most of the examined stations had at least satisfactory performance for three metrics for both daily and monthly time steps (Moriasi et al., 2007). Our results, therefore, support the findings of previous literature on the importance of representing water reservoir storage and operations in hydrological models (Boulange et al., 2021; Dang et al., 2020).

## 5 Discussion

### 5.1 Roles of Remote Sensing Imagery

The preceding analyses have clearly demonstrated that the proposed framework for extracting reservoir boundaries using the maximum water occurrence from the historical water map effectively improves the accuracy of the reservoir AEV relationships across all types of the reservoirs. When compared to the global reservoir database, our approach improves the “low‐fill” boundary extent and reduces the overestimation of the buffering approach around the GRanD reservoir boundaries. For small reservoirs that are <2 km^2^ (e.g., Sesan 4A), the proposed framework also produces AEV curves with NRMSE values of approximately 16% for Elevation‐Area and 6% for Elevation‐Volume. The accuracy of Elevation‐Volume estimates is better than Elevation‐Area estimates because public reservoir data at capacity was included in Equation 1. The upcoming Surface Water and Ocean Topography (SWOT) mission can provide two‐dimensional observations of water surface elevations and surface extent for water bodies with areas greater than 250 m × 250 m and with temporal resolution on the order of a few days, which will enable better estimations of Elevation‐Area curves (Durand et al., 2008; Lee et al., 2010).

The time series of estimated reservoir operation (i.e., elevations, storage volumes, and storage changes) generated by combining the AEV relationships and the Sentinel‐1‐derived reservoir areas agreed reasonably well for within‐year and over‐year reservoirs (when SR is usually ≥0.1), but relatively poorly for ROR reservoirs (SR typically <0.1). Due to the current limited low temporal sampling of freely available satellite imagery, daily storage changes can only be estimated by interpolation. Future studies can employ data fusion techniques to combine multi‐satellite missions (Ping et al., 2018; Shao et al., 2019) to improve these existing temporal resolutions. Nevertheless, it might still be challenging to obtain the estimated operation of ROR reservoirs with hydropeaking because they could have substantial flow changes at sub‐daily time scales. Future studies could examine more ROR reservoirs to confirm this finding.

Given the large gap in the number of reservoirs between global and local databases and limited availability of reservoir operation data, hydrological model setup over large river systems is still challenged by the lack of local regulation schemes. Previous studies (Table 1) rely on global geo‐referenced databases and do not quantitatively assess the capacity of satellite observations for monitoring reservoir operation across different reservoir operation behaviors. This limitation has thus prevented sufficient representation of human impacts in hydrological models. The framework proposed in this study can provide essential reservoir bathymetry for any local reservoirs and lakes and reservoir operation dynamics for within‐year and over‐year reservoirs. The information can help improve representation of existing local reservoirs in the global reservoir databases as well as representation of human impacts in hydrological models. In addition to the georeferenced locations and boundary extents provided by GRanD or GeoDAR, one of the most comprehensive global datasets, the World Register of Dams (WRD), maintained and updated by the International Commission of Large Dams (ICOLD; https://www.icold-cigb.org/), provides nearly 60,000 dams with more than 40 attributes. However, dam locations of WRD‐ICOLD databases are not georeferenced or accessible. The proposed framework, in integration with the participatory science approach of the Global River Obstruction Database (GROD) that identifies locations of dams through the use of cloud computing platform of GEE (Whittemore et al., 2020), could help supplement more detailed reservoir operation information for existing non‐georeferenced global reservoir database as well as non‐detailed georeferenced database like GlObal geOreferenced Database of Dams (GOODD; Mulligan et al., 2020).

### 5.2 Roles of Multi‐Basin Hydrological Model and IROS

The presence of reservoirs and lakes has been demonstrated to strongly affect the hydrologic model skill when predicting downstream streamflow. For ungauged reservoirs, with the increasing availability of satellite observations, the MB approach has been used to model reservoir outflows by combining hydrological models‐derived inflows and satellite‐based storage changes. However, as satellite‐based storage changes for ROR reservoirs were not accurately captured, using the MB approach for cascade systems with multiple ROR reservoirs would be subject to larger errors as found in Han et al. (2020). In this study, we used the same approach adopted in the HYPE model and named it the IROS. As IROS could model the actual reservoir storage capacity, this modeling approach could overcome the limitations of remote sensing imagery for ROR reservoirs, particularly at cascade reservoir systems. Therefore, it was shown that IROS could predict regulated streamflows satisfactorily for all types of reservoirs (i.e., within‐year, over‐year, ROR reservoirs) and across both individual reservoirs and cascade systems (Table 5). However, IROS did not perform well for cases where reservoirs have more than one sinusoidal cycle of water production (i.e., energy generation) within a year. A harmonic model with a flexible scheme to specify the number of cycles can improve the simulation of reservoir operation under conditions where there are multiple sinusoidal cycles of water production within a year and even within a day in the future. Nevertheless, rather than using generalized reservoir operation schemes across all reservoirs or the MB approach for all types of reservoirs, the IROS approach implemented here, in support with the remote sensing imagery, can improve the representation of human impacts in the hydrological models to provide more useful future predictions.

Under the supervision of the Department of Water Resources belonging to the Vietnam Ministry of Natural Resources and Environment, which since 2014 has been the agency responsible for developing and managing multi‐reservoir operation rules in Vietnam, reservoir operators have made significant attempts to follow the rules to avoid any lawsuits and fines (Nong Nghiep VN, 2016). Nevertheless, hydropeaking is still legally allowed in national energy planning policies for ROR reservoirs (Decision no. 478/MOIT, 2021) and exceptions for prioritizing energy production are still permitted to meet the country's energy demands and protect the grid systems. During 2016–2018, despite the environmental flows that were specified for each reservoir, consecutive days of significantly lower flows than the environmental flows were sometimes observed. The current policies discourage reservoir operators from investing in technologies to reduce the impacts on downstream communities and simultaneously improve energy generation and financial revenues (Ahmad & Hossain, 2019, 2020). Future studies can examine how different policy schemes and technologies can optimize reservoir operation, ensure energy production, and protect riverine ecosystems. Similarly, with the current proliferation of reservoir construction in areas upstream of Vietnam (e.g., in the upstream Mekong or Red River basins) and a consensus‐driven and non‐binding decision‐making process at the international transboundary of the Mekong River Basin, the cumulative impacts of cascade systems could be problematic as shown from operating examples of the Vietnamese reservoirs and previous studies (Hecht et al., 2019; Pokhrel et al., 2018). Not only could the hydrological impacts be dramatic, but the changing water (and associated sediment) budgets might disrupt the livelihoods of millions of people living in the Mekong and Red River delta (Bussi et al., 2021; Kondolf et al., 2018; Nguyen et al., 2021).

It is imperative to consider reservoir operations in highly regulated catchments to enable accurate estimation of impacts and to thereby develop appropriate actions for reducing negative impacts. Inclusion of all reservoirs and lakes, including small inland water bodies that are normally ignored in almost all global processes and cycles (Downing, 2010; Downing et al., 2006), can improve understanding of their important roles in processes such as climate regulation, carbon cycling, and biodiversity conservation (Arheimer et al., 2017; Smith et al., 2005; Tranvik et al., 2009). Additionally, a hydrological model with an improved representation of reservoir operations could provide useful information for many practical purposes. For example, such modeling could facilitate the attribution of individual extreme events to a particular cause and propose appropriate mitigation and adaptation plans for short‐term and long‐term management (Boulange et al., 2021; IFRC, 2020; Otto, 2016). Additionally, this type of modelling could be used with weather forecasting data to develop informed reservoir operations that optimize decisions on when to retain or release water. This important information can help sustain reservoir functions while reducing the negative impacts on downstream communities. As a result, both scientists and water managers could sustainably improve the monitoring and management of water resources and thus better address the United Nations Sustainable Development Goals (SDGs) as water is impeded in many SDGs (e.g., SDG6: Clean water and sanitation, SDG1: No Poverty, SDG2: Zero hunger, SDG3: Good health and well‐being, SDG7: Affordable and clean energy; United Nations, 2015).

## 6 Conclusion

As full representation of the human impacts in hydrological models is still unresolved due to our limited understanding of flow regulation schemes and insufficient reservoir data, this study highlights how satellite observations can be used to guide the model setup in highly regulated but poorly gauged transboundary river basins. Numerous prior studies have indicated the potentials of remote sensing imagery for monitoring reservoir operation within transboundary basins. However, few studies have investigated the extent to which reservoir releases can be inferred across different spatial scales and at varying temporal resolution. The proposed remote sensing imagery‐based reservoir operation framework has been successfully used to estimate AEV relationships reliably across all types of reservoirs. These AEV relationships, together with the interpolated daily reservoir surface areas, were combined to successfully create the rule curves and daily reservoir storage changes for within‐year and over‐year operating reservoirs. However, daily storage changes for ROR reservoirs with hydropeaking were found to be unsatisfactory due to the substantial flow changes within a day and the low temporal resolution of the remote sensing imagery used in this study. As most of recent satellite‐based reservoir operation studies have relied on the global‐scale geo‐referenced reservoir databases that often miss the local dams, the proposed framework can improve the representation of existing local reservoirs in the global reservoir database and thus the human impacts in hydrological models.

Although the dam operations can significantly change the hydrological responses and impacts of extreme events, previous approaches that have incorporated satellite observations for estimating streamflows still face difficulties within rivers that have cascading reservoir systems. The results presented in this paper demonstrate that incorporating IROS within a multi‐basin hydrological model and the use of remote sensing imagery‐based reservoir operation data can significantly improve the prediction of regulated streamflow downstream of both single reservoirs and reservoir cascades. As new dams and reservoirs continue to be planned and constructed, especially in largely ungauged basins in developing countries, improved representation of reservoir storage and operation in hydrological models is valuable in developing more useful adaptation measures to extreme events and reduce catastrophic impacts.

## Supporting information

Supporting Information S1

## Data Availability

The Greater Mekong‐HYPE model (GM‐HYPE) and related input and output data (e.g., merged precipitation, temperature, evaporation, streamflow, water levels, and reservoir data) is available from http://waterportal.vaci.org.vn/index.php. The HYPE model source codes are available from https://sourceforge.net/projects/hype/files/.

## References

[wrcr25884-bib-0001] AghaKouchak, A. , Mehran, A. , Norouzi, H. , & Behrangi, A. (2012). Systematic and random error components in satellite precipitation data sets. Geophysical Research Letters, 39(9). 10.1029/2012GL051592

[wrcr25884-bib-0002] Ahmad, S. K. , & Hossain, F. (2019). A generic data‐driven technique for forecasting of reservoir inflow: Application for hydropower maximization. Environmental Modelling & Software, 119, 147–165. 10.1016/j.envsoft.2019.06.008

[wrcr25884-bib-0003] Ahmad, S. K. , & Hossain, F. (2020). Maximizing energy production from hydropower dams using short‐term weather forecasts. Renewable Energy, 146, 1560–1577. 10.1016/j.renene.2019.07.126

[wrcr25884-bib-0004] Almeida, R. M. , Hamilton, S. K. , Rosi, E. J. , Barros, N. , Doria, C. R. C. , Flecker, A. S. , et al. (2020). Hydropeaking operations of two run‐of‐river mega‐dams alter downstream hydrology of the largest Amazon tributary. Frontiers in Environmental Science, 8. 10.3389/fenvs.2020.00120

[wrcr25884-bib-0005] Arheimer, B. , Donnelly, C. , & Lindström, G. (2017). Regulation of snow‐fed rivers affects flow regimes more than climate change. Nature Communications, 8(1), 62. 10.1038/s41467-017-00092-8 PMC5498529 28680129

[wrcr25884-bib-0006] Arheimer, B. , & Lindström, G. (2014). Electricity vs ecosystems—Understanding and predicting hydropower impact on Swedish river flow . Proceedings of the International Association of Hydrological Sciences, 364, 313–319. 10.5194/piahs-364-313-2014

[wrcr25884-bib-0007] Ashraf, F. B. , Haghighi, A. T. , Riml, J. , Alfredsen, K. , Koskela, J. J. , Kløve, B. , & Marttila, H. (2018). Changes in short term river flow regulation and hydropeaking in Nordic rivers. Scientific Reports, 8(1), 17232. 10.1038/s41598-018-35406-3 30467316 PMC6250702

[wrcr25884-bib-0008] Beck, H. E. , van Dijk, A. I. J. M. , Levizzani, V. , Schellekens, J. , Miralles, D. G. , Martens, B. , & de Roo, A. (2017). Mswep: 3‐hourly 0.25° global gridded precipitation (1979–2015) by merging gauge, satellite, and reanalysis data. Hydrology and Earth System Sciences, 21(1), 589–615. 10.5194/hess-21-589-2017

[wrcr25884-bib-0009] Beck, H. E. , Wood, E. F. , Pan, M. , Fisher, C. K. , Miralles, D. G. , van Dijk, A. I. J. M. , et al. (2019). MSWEP V2 global 3‐hourly 0.1° precipitation: Methodology and quantitative assessment. Bulletin of the American Meteorological Society, 100(3), 473–500. 10.1175/BAMS-D-17-0138.1

[wrcr25884-bib-0010] Biswas, N. K. , Hossain, F. , Bonnema, M. , Lee, H. , & Chishtie, F. (2021). Towards a Global Reservoir Assessment Tool for predicting hydrologic impacts and operating patterns of existing and planned reservoirs. Environmental Modelling & Software, 140, 105043. 10.1016/j.envsoft.2021.105043

[wrcr25884-bib-0011] Bonnema, M. , & Hossain, F. (2017). Inferring reservoir operating patterns across the Mekong Basin using only space observations. Water Resources Research, 53(5), 3791–3810. 10.1002/2016WR019978

[wrcr25884-bib-0012] Bonnema, M. , Sikder, S. , Miao, Y. , Chen, X. , Hossain, F. , Pervin, I. A. , et al. (2016). Understanding satellite‐based monthly‐to‐seasonal reservoir outflow estimation as a function of hydrologic controls. Water Resources Research, 52(5), 4095–4115. 10.1002/2015WR017830

[wrcr25884-bib-0013] Boulange, J. , Hanasaki, N. , Yamazaki, D. , & Pokhrel, Y. (2021). Role of dams in reducing global flood exposure under climate change. Nature Communications, 12(1), 417. 10.1038/s41467-020-20704-0 PMC7814128 33462241

[wrcr25884-bib-0014] Bussi, G. , Darby, S. E. , Whitehead, P. G. , Jin, L. , Dadson, S. J. , Voepel, H. E. , et al. (2021). Impact of dams and climate change on suspended sediment flux to the Mekong delta. The Science of the Total Environment, 755, 142468. 10.1016/j.scitotenv.2020.142468 33032131

[wrcr25884-bib-0015] C3S . (2017). Fifth generation of ECMWF atmospheric reanalyses of the global climate. Copernicus Climate Change Service Climate Data Store. Retrieved from https://cds.climate.copernicus.eu/cdsapp#!/home

[wrcr25884-bib-0016] Dang, T. D. , Chowdhury, A. F. M. K. , & Galelli, S. (2020). On the representation of water reservoir storage and operations in large‐scale hydrological models: Implications on model parameterization and climate change impact assessments. Hydrology and Earth System Sciences, 24(1), 397–416. 10.5194/hess-24-397-2020

[wrcr25884-bib-0017] De Vos, J. (2015). Non data‐driven reservoir outflow and storage simulations in hydrological models. Retreived from https://repository.tudelft.nl/islandora/object/uuid%3Ae68b26f2-1dad-41f3-81da-867b1d062458

[wrcr25884-bib-0018] Dinh, K. D. , Anh, T. N. , Nguyen, N. Y. , Bui, D. D. , & Srinivasan, R. (2020). Evaluation of grid‐based rainfall products and water balances over the Mekong River basin. Remote Sensing, 12(11), 1858. 10.3390/rs12111858

[wrcr25884-bib-0019] Döll, P. , Kaspar, F. , & Lehner, B. (2003). A global hydrological model for deriving water availability indicators: Model tuning and validation. Journal of Hydrology, 270(1), 105–134. 10.1016/S0022-1694(02)00283-4

[wrcr25884-bib-0020] Donchyts, G. , Schellekens, J. , Winsemius, H. , Eisemann, E. , & Van de Giesen, N. (2016). A 30 m resolution surface water mask including estimation of positional and thematic differences using Landsat 8, SRTM and OpenStreetMap: A case study in the Murray‐Darling basin, Australia. Remote Sensing, 8(5), 386. 10.3390/rs8050386

[wrcr25884-bib-0021] Downing, J. A. (2010). Emerging global role of small lakes and ponds: Little things mean a lot. Limnética, 29(1), 0009–0024. 10.23818/limn.29.02

[wrcr25884-bib-0022] Downing, J. A. , Prairie, Y. T. , Cole, J. J. , Duarte, C. M. , Tranvik, L. J. , Striegl, R. G. , et al. (2006). The global abundance and size distribution of lakes, ponds, and impoundments. Limnology & Oceanography, 51(5), 2388–2397. 10.4319/lo.2006.51.5.2388

[wrcr25884-bib-0023] Du, T. L. T. , Lee, H. , Bui, D. D. , Arheimer, B. , Li, H.‐Y. , Olsson, J. , et al. (2020). Streamflow prediction in “geopolitically ungauged” basins using satellite observations and regionalization at subcontinental scale. Journal of Hydrology, 588, 125016. 10.1016/j.jhydrol.2020.125016

[wrcr25884-bib-0024] Dubey, S. , Gupta, H. , Goyal, M. K. , & Joshi, N. (2021). Evaluation of precipitation datasets available on Google Earth engine over India. International Journal of Climatology, 41(10), 4844–4863. 10.1002/joc.7102

[wrcr25884-bib-0025] Durand, M. , Andreadis, K. M. , Alsdorf, D. E. , Lettenmaier, D. P. , Moller, D. , & Wilson, M. (2008). Estimation of bathymetric depth and slope from data assimilation of swath altimetry into a hydrodynamic model. Geophysical Research Letters, 35(20). 10.1029/2008GL034150

[wrcr25884-bib-0026] Farr, T. G. , Rosen, P. A. , Caro, E. , Crippen, R. , Duren, R. , Hensley, S. , et al. (2007). The Shuttle Radar Topography mission. Reviews of Geophysics, 45(2). 10.1029/2005RG000183

[wrcr25884-bib-0027] Funk, C. , Peterson, P. , Landsfeld, M. , Pedreros, D. , Verdin, J. , Shukla, S. , et al. (2015). The climate hazards infrared precipitation with stations—A new environmental record for monitoring extremes. Scientific Data, 2(1), 150066. 10.1038/sdata.2015.66 26646728 PMC4672685

[wrcr25884-bib-0028] Gao, H. , Birkett, C. , & Lettenmaier, D. P. (2012). Global monitoring of large reservoir storage from satellite remote sensing. Water Resources Research, 48(9). 10.1029/2012WR012063

[wrcr25884-bib-0029] Google Developers . (2020). Sentinel‐1 algorithms. Retrieved from https://developers.google.com/earth-engine/sentinel1

[wrcr25884-bib-0030] Gorelick, N. , Hancher, M. , Dixon, M. , Ilyushchenko, S. , Thau, D. , & Moore, R. (2017). Google Earth Engine: Planetary‐scale geospatial analysis for everyone. Remote Sensing of Environment, 202, 18–27. 10.1016/j.rse.2017.06.031

[wrcr25884-bib-0031] Grill, G. , Lehner, B. , Thieme, M. , Geenen, B. , Tickner, D. , Antonelli, F. , et al. (2019). Mapping the world’s free‐flowing rivers. Nature, 569(7755), 215–221. 10.1038/s41586-019-1111-9 31068722

[wrcr25884-bib-0032] Gupta, H. V. , Kling, H. , Yilmaz, K. K. , & Martinez, G. F. (2009). Decomposition of the mean squared error and NSE performance criteria: Implications for improving hydrological modelling. *Journal of Hydrology*, 377 (1), pp.80–91. 10.1016/j.jhydrol.2009.08.003

[wrcr25884-bib-0033] Han, Z. , Long, D. , Huang, Q. , Li, X. , Zhao, F. , & Wang, J. (2020). Improving reservoir outflow estimation for ungauged basins using satellite observations and a hydrological model. Water Resources Research, 56(9), e2020WR027590. 10.1029/2020WR027590

[wrcr25884-bib-0034] Hanasaki, N. , Kanae, S. , & Oki, T. (2006). A reservoir operation scheme for global river routing models. Journal of Hydrology, 327(1), 22–41. 10.1016/j.jhydrol.2005.11.011

[wrcr25884-bib-0035] Hecht, J. S. , Lacombe, G. , Arias, M. E. , Dang, T. D. , & Piman, T. (2019). Hydropower dams of the Mekong River basin: A review of their hydrological impacts. Journal of Hydrology, 568, 285–300. 10.1016/j.jhydrol.2018.10.045

[wrcr25884-bib-0036] Hoang, L. P. , van Vliet, M. T. H. , Kummu, M. , Lauri, H. , Koponen, J. , Supit, I. , et al. (2019). The Mekong’s future flows under multiple drivers: How climate change, hydropower developments and irrigation expansions drive hydrological changes. The Science of the Total Environment, 649, 601–609. 10.1016/j.scitotenv.2018.08.160 30176471

[wrcr25884-bib-0037] Huffman, G. J. , Bolvin, D. T. , Braithwaite, D. , Hsu, K. , Joyce, R. , Kidd, C. , et al. (2019). GPM IMERG final precipitation L3 half hourly 0.1 degree x 0.1 degree V06. Goddard Earth Sciences Data and Information Services Center. Retrieved from https://docserver.gesdisc.eosdis.nasa.gov/public/project/GPM/IMERG_ATBD_V06.pdf

[wrcr25884-bib-0038] IFRC . (2020). Operation update report Vietnam: Floods. Retrieved from https://www.ifrc.org/en/what-we-do/where-we-work/asia-pacific/red-cross-of-viet-nam/

[wrcr25884-bib-0039] Kim, D. , Lee, H. , Jung, H. C. , Hwang, E. , Hossain, F. , Bonnema, M. , et al. (2020). Monitoring river basin development and variation in water resources in transboundary Imjin River in North and South Korea using remote sensing. Remote Sensing, 12(1), 195. 10.3390/rs12010195

[wrcr25884-bib-0040] Kittikhoun, A. , & Staubli, D. M. (2018). Water diplomacy and conflict management in the Mekong: From rivalries to cooperation. Journal of Hydrology, 567, 654–667. 10.1016/j.jhydrol.2018.09.059

[wrcr25884-bib-0041] Klipsch, J. , & Hurst, M. (2007). HEC‐ResSim reservoir system simulation user’s manual version 3.0. U.S. Army Corps of Engineers. Retrieved from https://www.hec.usace.army.mil/Software/hec-ressim/documentation/HEC-ResSim_30_UsersManual.pdf

[wrcr25884-bib-0042] Knoben, W. J. M. , Freer, J. E. , & Woods, R. A. (2019). Technical note: Inherent benchmark or not? Comparing Nash–Sutcliffe and Kling–Gupta efficiency scores. Hydrology and Earth System Sciences, 23(10), 4323–4331. 10.5194/hess-23-4323-2019

[wrcr25884-bib-0043] Kondolf, G. M. , Schmitt, R. J. P. , Carling, P. , Darby, S. , Arias, M. , Bizzi, S. , et al. (2018). Changing sediment budget of the Mekong: Cumulative threats and management strategies for a large river basin. The Science of the Total Environment, 625, 114–134. 10.1016/j.scitotenv.2017.11.361 29288998

[wrcr25884-bib-0044] Kubota, T. , Shige, S. , Hashizume, H. , Aonashi, K. , Takahashi, N. , Seto, S. , et al. (2007). Global precipitation map using satellite‐borne microwave radiometers by the GSMaP Project: Production and validation. IEEE Transactions on Geoscience and Remote Sensing, 45(7), 2259–2275. 10.1109/TGRS.2007.895337

[wrcr25884-bib-0045] Lauri, H. , de Moel, H. , Ward, P. J. , Räsänen, T. A. , Keskinen, M. , & Kummu, M. (2012). Future changes in Mekong River hydrology: Impact of climate change and reservoir operation on discharge. Hydrology and Earth System Sciences, 16(12), 4603–4619. 10.5194/hess-16-4603-2012

[wrcr25884-bib-0046] Le, M.‐H. , Lakshmi, V. , Bolten, J. , & Bui, D. D. (2020). Adequacy of satellite‐derived precipitation estimate for hydrological modeling in Vietnam basins. Journal of Hydrology, 586, 124820. 10.1016/j.jhydrol.2020.124820

[wrcr25884-bib-0047] Lee, H. , Durand, M. , Jung, H. C. , Alsdorf, D. , Shum, C. K. , & Sheng, Y. (2010). Characterization of surface water storage changes in Arctic lakes using simulated SWOT measurements. International Journal of Remote Sensing, 31(14), 3931–3953. 10.1080/01431161.2010.483494

[wrcr25884-bib-0048] Lee, H. , Yuan, T. , Jung, H. C. , & Beighley, E. (2015). Mapping wetland water depths over the central Congo Basin using PALSAR ScanSAR, Envisat altimetry, and MODIS VCF data. Remote Sensing of Environment, 159, 70–79. 10.1016/j.rse.2014.11.030

[wrcr25884-bib-0049] Lehner, B. , Liermann, C. R. , Revenga, C. , Vörösmarty, C. , Fekete, B. , Crouzet, P. , et al. (2011). High‐resolution mapping of the world’s reservoirs and dams for sustainable river‐flow management. Frontiers in Ecology and the Environment, 9(9), 494–502. 10.1890/100125

[wrcr25884-bib-0050] Li, Y. , Gao, H. , Zhao, G. , & Tseng, K.‐H. (2020). A high‐resolution bathymetry dataset for global reservoirs using multi‐source satellite imagery and altimetry. Remote Sensing of Environment, 244, 111831. 10.1016/j.rse.2020.111831

[wrcr25884-bib-0051] Li, Z. , Tang, G. , Hong, Z. , Chen, M. , Gao, S. , Kirstetter, P. , et al. (2021). Two‐decades of GPM IMERG early and final run products intercomparison: Similarity and difference in climatology, rates, and extremes. Journal of Hydrology, 594, 125975. 10.1016/j.jhydrol.2021.125975

[wrcr25884-bib-0052] Lindström, G. (2016). Lake water levels for calibration of the S‐HYPE model. Hydrology Research, 47(4), 672–682. 10.2166/nh.2016.019

[wrcr25884-bib-0053] Lindström, G. , Pers, C. , Rosberg, J. , Strömqvist, J. , & Arheimer, B. (2010). Development and testing of the HYPE (Hydrological Predictions for the Environment) water quality model for different spatial scales. Hydrology Research, 41(3–4), 295–319. 10.2166/nh.2010.007

[wrcr25884-bib-0054] MARD . (2015). Report on dam safety. MARD.

[wrcr25884-bib-0055] Markert, K. N. , Markert, A. M. , Mayer, T. , Nauman, C. , Haag, A. , Poortinga, A. , et al. (2020). Comparing Sentinel‐1 surface water mapping algorithms and radiometric terrain correction processing in Southeast Asia utilizing Google Earth engine. Remote Sensing, 12(15), 2469. 10.3390/rs12152469

[wrcr25884-bib-0056] Masaki, Y. , Hanasaki, N. , Biemans, H. , Schmied, H. M. , Tang, Q. , Wada, Y. , et al. (2017). Intercomparison of global river discharge simulations focusing on dam operation—M6ultiple models analysis in two case‐study river basins, Missouri–Mississippi and Green–Colorado. Environmental Research Letters, 12(5), 055002. 10.1088/1748-9326/aa57a8 30377438 PMC6204261

[wrcr25884-bib-0057] McManamay, R. A. , Oigbokie, C. O. , Kao, S.‐C. , & Bevelhimer, M. S. (2016). Classification of US hydropower dams by their modes of operation. River Research and Applications, 32(7), 1450–1468. 10.1002/rra.3004

[wrcr25884-bib-0058] MOIT . (2021). Quyết định số 478/QĐ‐BCT ‐ Về Việc Dịch Chuyển Giờ Phát điện Cao Điểm Các Nhà Máy Thủy điện Nhỏ. (in English: Decision no. 478/QĐ‐BCT ‐ Regarding Shifting Peak Power Generation Time for Small Hydropower Plants) Retrieved from https://thuvienphapluat.vn/van-ban/Tai-nguyen-Moi-truong/Quyet-dinh-478-QD-BCT-2021-dich-chuyen-gio-phat-dien-cao-diem-nha-may-thuy-dien-nho-465743.aspx

[wrcr25884-bib-0059] MONRE . (2017). Thông tư 47/2017/TT‐BTNMT ‐ Quy định Về Giám Sát Khai Thác, Sử Dụng Tài Nguyên Nước. (in English: Circular no. 47/2017/TT‐BTNMT ‐Regulations on monitoring exploitation and use of Water Resources). MONRE. Retrieved from https://thuvienphapluat.vn/van-ban/Tai-nguyen-Moi-truong/Thong-tu-47-2017-TT-BTNMT-quy-dinh-giam-sat-khai-thac-su-dung-tai-nguyen-nuoc-325036.aspx

[wrcr25884-bib-0060] MONRE . (2020). Report on water resources condition, reservoir operation and water deficit in all river basins. MONRE.

[wrcr25884-bib-0061] Moriasi, D. N. , Arnold, J. G. , Van Liew, M. W. , Bingner, R. L. , Harmel, R. D. , & Veith, T. L. (2007). Model evaluation guidelines for systematic quantification of accuracy in watershed simulations. Transactions of the ASABE, 50(3), 885–900. 10.13031/2013.23153

[wrcr25884-bib-0062] Mulligan, M. , van Soesbergen, A. , & Sáenz, L. (2020). GOODD, a global dataset of more than 38,000 georeferenced dams. Scientific Data, 7(1), 31. 10.1038/s41597-020-0362-5 31964896 PMC6972789

[wrcr25884-bib-0063] Nash, J. E. , & Sutcliffe, J. V. (1970). River flow forecasting through conceptual models part I—A discussion of principles. Journal of Hydrology, 10(3), 282–290. 10.1016/0022-1694(70)90255-6

[wrcr25884-bib-0064] Nazemi, A. , & Wheater, H. S. (2015). On inclusion of water resource management in Earth system models**—**Part 1: Problem definition and representation of water demand. Hydrology and Earth System Sciences, 19(1), 33–61. 10.5194/hess-19-33-2015

[wrcr25884-bib-0065] Nguyen, D. V. , Fan, D. , Van Vuong, B. , & Lan, T. D. (2021). Sediment budget and morphological change in the Red River Delta under increasing human interferences. Marine Geology, 431, 106379. 10.1016/j.margeo.2020.106379

[wrcr25884-bib-0066] Nong Nghiep, V. N. (2016). Thủy điện Đăk Mi 4 bị xử phạt 810 triệu đồng (in English: Dak Ni 4 Hydropower Plant was fined 810 million VND). Retrieved from https://nongnghiep.vn/thuy-dien-dak-mi-4-bi-xu-phat-810-trieu-dong-d160647.html

[wrcr25884-bib-0067] Otsu, N. (1979). A threshold selection method from gray‐level histograms. IEEE Transactions on Systems, Man, and Cybernetics, 9, 62–66. 10.1109/TSMC.1979.4310076

[wrcr25884-bib-0068] Otto, F. E. L. (2016). The art of attribution. Nature Climate Change, 6(4), 342–343. 10.1038/nclimate2971

[wrcr25884-bib-0069] Park, E. , Merino, E. , Lewis, Q. W. , Lindsey, E. O. , & Yang, X. (2020). A pathway to the automated global assessment of water level in reservoirs with synthetic aperture radar (SAR). Remote Sensing, 12(8), 1353. 10.3390/rs12081353

[wrcr25884-bib-0070] Pechlivanidis, I. G. , & Arheimer, B. (2015). Large‐scale hydrological modelling by using modified PUB recommendations: The India‐HYPE case. Hydrology and Earth System Sciences, 19(11), 4559–4579. 10.5194/hess-19-4559-2015

[wrcr25884-bib-0071] Pekel, J.‐F. , Cottam, A. , Gorelick, N. , & Belward, A. S. (2016). High‐resolution mapping of global surface water and its long‐term changes. Nature, 540(7633), 418–422. 10.1038/nature20584 27926733

[wrcr25884-bib-0072] Pham, H. T. (2015). Dilemmas of hydropower development in Vietnam: Between dam‐induced displacement and sustainable development. Eburon Uitgeverij B.V.

[wrcr25884-bib-0073] Ping, B. , Meng, Y. , & Su, F. (2018). An enhanced linear spatio‐temporal fusion method for blending Landsat and MODIS data to synthesize Landsat‐like imagery. Remote Sensing, 10(6), 881. 10.3390/rs10060881

[wrcr25884-bib-0074] Pokhrel, Y. , Shin, S. , Lin, Z. , Yamazaki, D. , & Qi, J. (2018). Potential disruption of flood dynamics in the lower Mekong River basin due to upstream flow regulation. Scientific Reports, 8(1), 17767. 10.1038/s41598-018-35823-4 30532063 PMC6288158

[wrcr25884-bib-0075] Pushpalatha, R. , Perrin, C. , Moine, N. L. , & Andréassian, V. (2012). A review of efficiency criteria suitable for evaluating low‐flow simulations. Journal of Hydrology, 420–421, 171–182. 10.1016/j.jhydrol.2011.11.055

[wrcr25884-bib-0076] Rodríguez, E. , Morris, C. S. , & Belz, J. E. (2006). A global assessment of the SRTM performance. Photogrammetric Engineering & Remote Sensing, 72(3), 249–260. 10.14358/PERS.72.3.249

[wrcr25884-bib-0077] Roy, D. P. , Wulder, M. A. , Loveland, T. R. , C.e., W. , Allen, R. G. , Anderson, M. C. , et al. (2014). Landsat‐8: Science and product vision for terrestrial global change research. Remote Sensing of Environment, 145, 154–172. 10.1016/j.rse.2014.02.001

[wrcr25884-bib-0078] Saha, S. , Moorthi, S. , Wu, X. , Wang, J. , Nadiga, S. , Tripp, P. , et al. (2011). NCEP Climate Forecast System Version 2 (CFSv2) 6‐hourly Products (Vol. 10, p. D61C1TXF). Research Data Archive at the National Center for Atmospheric Research, Computational and Information Systems Laboratory.

[wrcr25884-bib-0079] Shao, Z. , Cai, J. , Fu, P. , Hu, L. , & Liu, T. (2019). Deep learning‐based fusion of Landsat‐8 and Sentinel‐2 images for a harmonized surface reflectance product. Remote Sensing of Environment, 235, 111425. 10.1016/j.rse.2019.111425

[wrcr25884-bib-0080] Sheffield, J. , Wood, E. F. , Pan, M. , Beck, H. , Coccia, G. , Serrat‐Capdevila, A. , & Verbist, K. (2018). Satellite remote sensing for water resources management: Potential for supporting sustainable development in data‐poor regions. Water Resources Research, 54(12), 9724–9758. 10.1029/2017WR022437

[wrcr25884-bib-0081] Shiklomanov, A. I. , Lammers, R. B. , & Vörösmarty, C. J. (2002). Widespread decline in hydrological monitoring threatens Pan‐Arctic Research. Eos, 83(2), 13–17. 10.1029/2002EO000007

[wrcr25884-bib-0082] Shin, S. , Pokhrel, Y. , & Miguez‐Macho, G. (2019). High‐resolution modeling of reservoir release and storage dynamics at the continental scale. Water Resources Research, 55(1), 787–810. 10.1029/2018WR023025

[wrcr25884-bib-0083] Smith, L. C. , Sheng, Y. , MacDonald, G. M. , & Hinzman, L. D. (2005). Disappearing Arctic lakes. Science, 308(5727), 1429. 10.1126/science.1108142 15933192

[wrcr25884-bib-0084] Tang, L. , Tian, Y. , Yan, F. , & Habib, E. (2015). An improved procedure for the validation of satellite‐based precipitation estimates. Atmospheric Research, 163, 61–73. 10.1016/j.atmosres.2014.12.016

[wrcr25884-bib-0085] Terink, W. , Lutz, A. F. , Simons, G. W. H. , Immerzeel, W. W. , & Droogers, P. (2015). SPHY v2.0: Spatial processes in HYdrology. Geoscientific Model Development, 8(7), 2009–2034. 10.5194/gmd-8-2009-2015

[wrcr25884-bib-0086] Thu, H. N. , & Wehn, U. (2016). Data sharing in international transboundary contexts: The Vietnamese perspective on data sharing in the Lower Mekong Basin. Journal of Hydrology, 536, 351–364. 10.1016/j.jhydrol.2016.02.035

[wrcr25884-bib-0087] Tranvik, L. J. , Downing, J. A. , Cotner, J. B. , Loiselle, S. A. , Striegl, R. G. , Ballatore, T. J. , et al. (2009). Lakes and reservoirs as regulators of carbon cycling and climate. Limnology & Oceanography, 54(6part2), 2298–2314. 10.4319/lo.2009.54.6_part_2.2298

[wrcr25884-bib-0088] United Nations . (2015). Transforming our world: The 2030 Agenda for sustainable development. United Nations. Retrieved from https://sdgs.un.org/sites/default/files/publications/21252030%20Agenda%20for%20Sustainable%20Development%20web.pdf

[wrcr25884-bib-0089] Vietnamese Government . (2013). Nghị định 201/2013/NĐ‐CP—Quy định Chi Tiết thi Hành Một số Điều Của Luật Tài Nguyên Nước. (in English: Decree no. 201/2013/NĐ‐CP ‐ Detailed Regulations for the Implementation of Certain Articles of the Law on Water Resources) Retrieved from https://thuvienphapluat.vn/van-ban/Tai-nguyen-Moi-truong/Nghi-dinh-201-2013-ND-CP-huong-dan-Luat-tai-nguyen-nuoc-214786.aspx

[wrcr25884-bib-0090] Vietnamese Government . (2018). Nghị định 114/2018/NĐ‐CP Về Quản Lý an Toàn Đập, Hồ Chứa Nước. (in English: Decree no. 114/2018/NĐ‐CP, 2018 on Safety Management of Dams and Reservoirs). Retrieved from https://thuvienphapluat.vn/van-ban/Xay-dung-Do-thi/Nghi-dinh-114-2018-ND-CP-quan-ly-an-toan-dap-ho-chua-nuoc-393268.aspx

[wrcr25884-bib-0091] Vogel, R. M. , Lane, M. , Ravindiran Ranjith, S. , & Kirshen, P. (1999). Storage reservoir behavior in the United States. Journal of Water Resources Planning and Management, 125(5), 245–254. 10.1061/(ASCE)0733-9496(1999)125:5(245)

[wrcr25884-bib-0092] Vogel, R. M. , Sieber, J. , Archfield, S. A. , Smith, M. P. , Apse, C. D. , & Huber‐Lee, A. (2007). Relations among storage, yield, and instream flow. Water Resources Research, 43(5). 10.1029/2006WR005226

[wrcr25884-bib-0093] Wang, J. , Walter, B. A. , Yao, F. , Song, C. , Ding, M. , Maroof, A. S. , et al. (2021). GeoDAR: Georeferenced global dam and reservoir dataset for bridging attributes and geolocations. Earth System Science Data Discussions, 1–52. 10.5194/essd-2021-58

[wrcr25884-bib-0094] Weekley, D. , & Li, X. (2021). Tracking lake surface elevations with proportional hypsometric relationships, Landsat imagery, and multiple DEMs. Water Resources Research, 57(1), e2020WR027666. 10.1029/2020WR027666

[wrcr25884-bib-0095] Whittemore, A. , Ross, M. R. V. , Dolan, W. , Langhorst, T. , Yang, X. , Pawar, S. , et al. (2020). A participatory science approach to expanding instream infrastructure inventories. Earth’s Future, 8(11), e2020EF001558. 10.1029/2020EF001558

[wrcr25884-bib-0096] Wisser, D. , Fekete, B. M. , Vörösmarty, C. J. , & Schumann, A. H. (2010). Reconstructing 20th century global hydrography: A contribution to the global terrestrial network‐ hydrology (GTN‐H). Hydrology and Earth System Sciences, 14(1), 1–24. 10.5194/hess-14-1-2010

[wrcr25884-bib-0097] WLE Greater Mekong . (n.d.). WLE Greater Mekong dams observatory. WLE Mekong. Retrieved from https://wle-mekong.cgiar.org/changes/our-research/greater-mekong-dams-observatory/

[wrcr25884-bib-0098] World Bank . (2015). Project information document (concept stage)‐Vietnam dam rehabilitation and safety improvement project‐P152309. World Bank Group.

[wrcr25884-bib-0099] Xu, H. (2006). Modification of normalised difference water index (NDWI) to enhance open water features in remotely sensed imagery. International Journal of Remote Sensing, 27(14), 3025–3033. 10.1080/01431160600589179

[wrcr25884-bib-0100] Zajac, Z. , Revilla‐Romero, B. , Salamon, P. , Burek, P. , Hirpa, F. A. , & Beck, H. (2017). The impact of lake and reservoir parameterization on global streamflow simulation. Journal of Hydrology, 548, 552–568. 10.1016/j.jhydrol.2017.03.022 28649141 PMC5473175

[wrcr25884-bib-0101] Zhang, S. , & Gao, H. (2020). Using the digital elevation model (DEM) to improve the spatial coverage of the MODIS based reservoir monitoring network in South Asia. Remote Sensing, 12(5), 745. 10.3390/rs12050745

[wrcr25884-bib-0102] Zhao, G. , & Gao, H. (2018). Automatic correction of contaminated images for assessment of reservoir surface area dynamics. Geophysical Research Letters, 45(12), 6092–6099. 10.1029/2018GL078343 35095126 PMC8793793

[wrcr25884-bib-0103] Zhao, G. , Gao, H. , Naz, B. S. , Kao, S.‐C. , & Voisin, N. (2016). Integrating a reservoir regulation scheme into a spatially distributed hydrological model. Advances in Water Resources, 98, 16–31. 10.1016/j.advwatres.2016.10.014

[wrcr25884-bib-0104] Oudin, L. , Andréassian, V. , Mathevet, T. , Perrin, C. , & Michel, C. (2006). Dynamic averaging of rainfall‐runoff model simulations from complementary model parameterizations. Water Resources Research, 42(7). 10.1029/2005WR004636

