# Supplementary material for: Streamflow Prediction in Highly Regulated, Transboundary Watersheds Using Multi‐Basin Modeling and Remote Sensing Imagery
Source: Water Resour Res. 2022 Mar 24;58(3):e2021WR031191. doi: 10.1029/2021WR031191 (PMC9286455; doi:10.1029/2021WR031191)
Supplement: Supplementary file 1 — Supporting Information S1 [file WRCR-58-0-s001.docx]

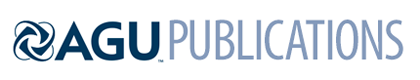


*Water Resources Research*

Supporting Information for

**Streamflow Prediction in Highly Regulated, Transboundary Watersheds Using Multi-Basin Modelling and Remote Sensing Imagery**

**Tien L.T. Du^1,2^, Hyongki Lee^1^, Duong D. Bui^3*^, L. Phil Graham^4^, Stephen D. Darby^5^, Ilias G. Pechlivanidis^4^, Julian Leyland^5^, Nishan K. Biswas^6^, Gyewoon Choi^7^, Okke Batelaan^8^, Thao T.P Bui^9^, Son K. Do^1^, Tinh V. Tran^10^, Hoa Thi Nguyen^11^, Euiho Hwang^12^**

^1^Department of Civil and Environmental Engineering, University of Houston, Houston, TX, USA.

^2^Danang Institute for Socio-Economic Development, Da Nang, Vietnam.

^3^National Center for Water Resources Planning and Investigation, Ministry of Natural Resources and Environment, Hanoi, Vietnam.

^4^Swedish Meteorological and Hydrological Institute (SMHI), Norrköping, Sweden.

^5^School of Geography and Environmental Science, University of Southampton, Southampton, UK.

^6^NASA Goodard Space Flight Center, Greenbelt, MD, USA.

^7^International Center for Urban Water Hydroinformatics Research & Innovation, Republic of Korea.

^8^Flinders University, Adelaide, Australia.

^9^Department of Civil and Environmental Engineering, Tokyo Metropolitan University, Tokyo, Japan.

^10^Department of Water Resources, Hanoi University of Natural Resource and Environment (HUNRE), Hanoi, Vietnam.

^11^Faculty of Environment, Hanoi University of Mining and Geology (HUGM), Hanoi, Vietnam.

^12^Water Resources Satellite Research Center, K-Water Institute, K-water, Daejeon, Republic of Korea.

*Corresponding author: Duong Du Bui ([duongdubui@gmail.com](mailto:duongdubui@gmail.com)).

# **Contents of this file**

Appendix A

Tables S1 to S7

Figure S1 to S2

References

# **Introduction**

The supporting information provides the content of:

- Appendix A. Mass balance approach for estimating averaged outflows to calibrate IROS parameters.
- Table S1. Description of validated gauged reservoirs and streamflow stations in the Greater Mekong region.
- Table S2. Performance metrics used in the study
- Table S3. Statistical evaluation of Sentinel-1-derived reservoir surface areas, elevations and storage volumes and storage changes. Medians with their standard deviations shown in the parentheses. Unit is provided for median $RMSE$. Mcm stands for million cubic meter.
- Table S4. Statistical evaluation of simulated inflows of the first order reservoirs in each cascade system. Medians with their standard deviations shown in the parentheses.
- Table S5. Statistical evaluation of IROS-based $\Delta S$ for both gauged and ungauged reservoirs. Medians with their standard deviations shown in the parentheses. Unit is provided for median $RMSE$. Mcm for million cubic meter.
- Table S6. Statistical evaluation of MB-based reservoir outflows. Medians with their standard deviations shown in the parentheses. Unit is provided for median $RMSE$.
- Table S7. Statistical evaluation of IROS-based reservoir outflows. Medians with their standard deviations shown in the parentheses. Unit is provided for median $RMSE$.
- Figure S1. Sentine1-derived time series of the reservoir surface areas, elevations and storage volumes for three sample reservoirs compared with observed (Obs) data. Tuyen Quang is an over-year reservoir, Son La is a within-year reservoir, and Buon Kop is an ROR reservoir. ‘Interp.’ denotes interpolated data. ‘Original’ denotes non-interpolated data. ‘S1’ denotes Sentinel-1. Locations of the reservoirs within the Greater Mekong study region are shown in Figure 1.
- Figure S2. The IROS-based simulated storage changes (IROS $\Delta S$) compared with Sentinel-1-derived (S1 $\Delta S$) and observed (Obs $\Delta S$) storage changes.

Appendix A. Mass-balance approach

Using the classic MB approach, reservoir outflow ($Q_{out}$) can be estimated by subtracting evaporation ($E$) and reservoir storage changes ($\Delta S$) from inflow ($Q_{in}$) (Eq.1a). Here, therefore, $Q_{in}$ and $E$ were obtained from GM-HYPE v.1.4 (see Section 3.2) whereas $\Delta S$ was obtained from remotely sensed data (Section 3.1). Since daily $\Delta S$ can only be obtained by interpolating low temporal samplings of Landsat-8 or Sentinel-1, estimation errors cannot be avoided. Accordingly, when $\frac{\Delta S}{t}$ ($t$ here is the daily time employed in this study) is greater than $(Q_{in}-E)$, any unrealistic negative outflow as a result was set to be the missing flow (*NaN*) for that day (Eq.1b). Since the objective of this step is to calibrate IROS parameters and validate IROS-based outflows, the missing flow approach was used to reduce error propagation.

$Q_{out}= \left\{ \begin{aligned} Q_{in}-E-\frac{\Delta S}{t} \text{ } \text{ }\left( \text{a} \right)\text{ if }\left( Q_{in}-E \right)\geq\frac{\Delta S}{t} \\ NaN\text{ (b) if }\left( Q_{in}-E \right)<\frac{\Delta S}{t} (\text{for testing in this study}) \\ \end{aligned} \right.$ (1)

Table S1. Description of validated gauged reservoirs and streamflow stations in the Greater Mekong region. Data sources are as follows: (i) gauged reservoirs: Vietnam Electricity (EVN) and the Department of Water Resources Management from Vietnam Ministry of Natural Resources and Environment, (ii) ungauged reservoirs: Global Reservoir and Dam database (GRanD) and Mekong Dam Observatory (WLE Greater Mekong); (iii) gauged hydrological stations in Vietnam: Vietnam Meteorological and Hydrological Administration and (iv) gauged hydrological stations outside Vietnam: Asian Disaster Preparedness Center (SERVIR-Mekong hub).

| **No** | **Name** | **River** | **River basin** | **Order within cascade systems** | **Country** | **GRanD**  **ID** | **Catchment**  **area**  **(km^2^)** | **Year**  **of**  **commission** | **Operation**  **mode** | **Purposes** | **Average annual inflow (m^3^/s)** | **Total capacity (MCM)** | **Storage Ratio** |
| --- | --- | --- | --- | --- | --- | --- | --- | --- | --- | --- | --- | --- | --- |
| **Gauged Reservoirs** | | | | | | | | | | | | | |
| 1. | Tuyen Quang | Gam Tributary | Red-Thai Binh | 1^st^ | VN | 7209 | 14972 | 2008 | Over-year | Multi  purpose | 293.527 | 2260 | 0.244 |
| 2. | Thac Ba | Lo  Tributary | Red-Thai Binh | 1^st^ | VN | 5779 | 6430 | 1971 | Over-year | Multi-purpose | 182.191 | 2940 | 0.512 |
| 3. | Ban Chat | Nam Mu  Tributary | Red-Thai Binh | 1^st^ | VN | 7154 | 2067 | 2013 | Within-year | Hydropower | 90.904 | 2137.7 | 0.746 |
| 4. | Huoi Quang | Nam Mu  Tributary | Red-Thai Binh | 2^nd^ | VN | 7165 | 2965 | 2016 | ROR | Hydropower | 120.324 | 184.2 | 0.049 |
| 5. | Lai Chau | Da  Tributary | Red-Thai Binh | 5^th^ | VN | 7166 | 25529 | 2016 | Within-year | Multi-purpose | 692.257 | 1215.1 | 0.056 |
| 6. | Son La | Da  Tributary | Red-Thai Binh | 6^th^ | VN | 7204 | 43760 | 2012 | Within-year | Multi-purpose | 1337.047 | 9260 | 0.22 |
| 7. | Hoa Binh | Da  Tributary | Red-Thai Binh | 7^th^ | VN | 5782 | 51700 | 1994 | Within-year | Multi-purpose | 1522.101 | 9862 | 0.205 |
| 8. | Ban Ve | Nam Non  Tributary | Ca | 1^st^ | VN | 7155 | 8700 | 2010 | Within-year | Multi-purpose | 143.954 | 1834.6 | 0.404 |
| 9. | Khe Bo | Ca  Mainstream | Ca | 2^nd^ | VN | NA | 14300 | 2013 | ROR | Hydropower | 306.087 | 97.8 | 0.01 |
| 10. | Ham Thuan | La Nga  Tributary | Dong Nai | 1^st^ | VN | 5801 | 1280 | 2001 | Within-year, diverted to Da Mi | Multi-purpose | 46.453 | 695.23 | 0.475 |
| 11. | Da Mi | La Nga  Tributary | Dong Nai | 2^nd^ | VN | 5802 | 110 | 2001 | ROR | Hydropower | 50.44* | 140.8 | 0.09 |
| 12. | Pleikrong | Sesan  Tributary | 2S | 1^st^ | VN | 7201 | 3216 | 2010 | Within-year | Multi-purpose | 135.919 | 1048.7 | 0.245 |
| 13. | Yali | Sesan  Tributary | 2S | 2^nd^ | VN | 5798 | 7455 | 2000 | Within-year | Multi-purpose | 271.351 | 1037 | 0.121 |
| 14. | Sesan 3 | Sesan  Tributary | 2S | 3^rd^ | VN | NA | 7788 | 2007 | ROR | Hydropower | 287.175 | 86.7 | 0.01 |
| 15. | Sesan 3A | Sesan  Tributary | 2S | 4^th^ | VN | NA | 8084 | 2007 | ROR | Hydropower | 301.241 | 80.6 | 0.008 |
| 16. | Sesan 4 | Sesan  Tributary | 2S | 5^th^ | VN | 7203 | 9326 | 2009 | Within-year | Multi-purpose | 358.89 | 893.3 | 0.079 |
| 17. | Sesan 4A | Sesan  Tributary | 2S | 6^th^ | VN | NA | 9368 | 2011 | ROR | Hydropower | 360.875 | 13.1 | 0.001 |
| 18. | Buon Tua Srah | Srepok  Tributary | 2S | 1^st^ | VN | 7158 | 2930 | 2011 | Within-year | Multi-purpose | 107.043 | 786.9 | 0.233 |
| 19. | Buon Kop | Srepok  Tributary | 2S | 2^nd^ | VN | 7159 | 7980 | 2010 | ROR | Hydropower | 241.546 | 63.24 | 0.008 |
| 20. | Srepok 3 | Srepok  Tributary | 2S | 3^rd^ | VN | NA | 9410 | 2010 | ROR | Hydropower | 276.59 | 218.99 | 0.025 |
| 21. | Srepok 4 | Srepok  Tributary | 2S | 4^th^ | VN | NA | 9344 | 2011 | ROR | Hydropower | 279.787 | 25.94 | 0.003 |
| **Ungauged reservoirs** | | | | | | | | | | | | | |
| 1. | Nansha | Honghe | Red-ThaiBinh | 1st | China | 7228 | 28842 | 2008 |  | Hydropower | 284.50 | 212 | 0.024 |
| 2. | Madushan | Hong | Red-ThaiBinh | 2nd | China | 7261 | 31336 | 2011 |  | Hydropower | 307.06 | 551 | 0.057 |
| 3. | Puxiqiao | Amo | Red-ThaiBinh | 1st | China | 7286 | 4161 | 2013 |  | Hydropower | 55.80 | 521 | 0.296 |
| 4. | Sinanjiang | Sinan | Red-ThaiBinh | 1st | China | 7247 | 1594 | 2008 |  | Hydropower | 29.73 | 271 | 0.289 |
| 5. | Yayangshan | Babian | Red-ThaiBinh | 1st | China | 7278 | 6141 | 2006 |  | Hydropower | 71.01 | 247 | 0.110 |
| 6. | Shimenkan | Lixian | Red-ThaiBinh | 2nd | China | 7243 | 6441 | 2010 |  | Hydropower | 77.54 | 197 | 0.081 |
| 7. | Longma | Lixian | Red-ThaiBinh | 1st | China | 7195 | 8777 | 2007 |  | Hydropower | 143.67 | 590 | 0.130 |
| 8. | Jupudu | Lixian | Red-ThaiBinh | 3rd | China | 7185 | 16093 | 2007 |  | Hydropower | 261.94 | 174 | 0.021 |
| 9. | Gelantan | Lixian | Red-ThaiBinh | 4th | China | 7085 | 17262 | 2009 |  | Hydropower | 277.87 | 409 | 0.047 |
| 10. | Nalan | Tentiaojiang | Red-ThaiBinh | 1st | China | 7227 | 2836 | 2005 |  | Hydropower | 71.88 | 286 | 0.126 |
| 11. | Malutang | Panlong | Red-ThaiBinh | 1st | China | 7220 | 5085 | 2009 |  | Hydropower | 74.92 | 546 | 0.231 |
| 12. | Wunonglong | LanCang | Mekong | 1st | China | NA | 85900 | 2018 |  | Hydropower | 457.00 | 284 | 0.020 |
| 13. | Lidi | LanCang | Mekong | 2nd | China | NA | NA | 2019 |  | Hydropower | 486.74 | 75 | 0.005 |
| 14. | HuangDeng | LanCang | Mekong | 3rd | China | NA | NA | 2017 |  | Hydropower | 549.64 | 1613 | 0.093 |
| 15. | DaHuaQiao | LanCang | Mekong | 4th | China | NA | 92600 | 2018 |  | Hydropower | 555.98 | 293 | 0.017 |
| 16 | MiaoWei | LanCang | Mekong | 5th | China | NA | 93900 | 2016 |  | Hydropower | 569.11 | 660 | 0.037 |
| 17. | Gongguoqiao | LanCang | Mekong | 6th | China | 7087 | 90771 | 2012 |  | Hydropower | 817.58 | 316 | 0.012 |
| 18. | Xiaowan | LanCang | Mekong | 7th | China | 7284 | 106952 | 2012 |  | Hydropower | 817.58 | 15043 | 0.583 |
| 19. | Manwan | LanCang | Mekong | 8th | China | 5117 | 108067 | 1995 |  | Hydropower | 822.95 | 920 | 0.035 |
| 20. | Dachaoshan | LanCang | Mekong | 9th | China | 7037 | 113875 | 2002 |  | Hydropower | 898.90 | 940 | 0.033 |
| 21. | Nuozhadu | LanCang | Mekong | 10th | China | 7232 | 137909 | 2014 |  | Hydropower | 898.90 | 23703 | 0.836 |
| 22. | Jinghong | LanCang | Mekong | 11th | China | 7181 | 142559 | 2009 |  | Hydropower | 1444.60 | 1139 | 0.025 |
| 23. | Nam Ngum 5 | Nam Ngum | Mekong | 1st | Laos | 7002 | 409 | 2012 |  | Hydropower | 13.32 | 314 | 0.748 |
| 24. | Nam Ngum 2 | Nam Ngum | Mekong | 2nd | Laos | 7001 | 5698 | 2011 |  | Hydropower | 175.79 | 4230 | 0.763 |
| 25. | Nam Leuk | Nam Leuk | Mekong | 1st | Laos | 5138 | 276 | 2000 |  | Hydropower | 31.70 | 185 | 0.185 |
| 26. | Nam Ngum | Nam Ngum | Mekong | 3rd | Laos | 5136 | 8308 | 1972 |  | Hydropower | 282.73 | 7030 | 0.788 |
| 27. | Nam Mang 3 | Nam Gnong | Mekong | 1st | Laos | 7000 | 62 | 2004 |  | Hydropower | 200.00 | 494 | 0.078 |
| 28. | Nam Theun 2 | Nam Theun | Mekong | 1st | Laos | 6999 | 3945 | 2010 |  | Hydropower | 197.60 | 3210 | 0.515 |
| 29. | Xe Kaman 3 | Xe Kaman | Mekong | 1st | Laos | 7004 | 677 | 2013 |  | Hydropower | 45.20 | 141 | 0.099 |
| 30. | Houayho | Hoauyho | Mekong | 1st | Laos | 5797 | 174 | 1999 |  | Hydropower | 7.30 | 649 | 2.819 |
| 31. | Xe Kaman 1 | Xe Kaman | Mekong | 2nd | Laos | 7003 | 3658 | 2015 |  | Hydropower | 168.20 | 3210 | 0.605 |
| 32. | Nam_Oun | Khong | Mekong | 1st | Thailand | 5143 | 1087 | NA |  | Irrigation | 26.63 | 520 | 0.619 |
| 33. | Nam Pung | Nam Pung | Mekong | 1st | Thailand | 5148 | 286 | 1965 |  | Hydropower | 6.40 | 165.5 | 0.820 |
| 34. | Nong_Han_Lake | NA | Mekong | 1st | Thailand | 5147 | 1650 | NA |  | Recreation | 39.50 | 1874.4 | 1.505 |
| 35. | Chulabhorn | Nam Phrom | Mekong | 1st | Thailand | 5151 | 516 | 1972 |  | Hydropower | 4.30 | 188 | 1.386 |
| 36. | Huai Kum | Nam Phrom | Mekong | 2nd | Thailand | 5152 | 803 | 1982 |  | Hydropower | 8.70 | 22.8 | 0.083 |
| 37. | Ubol_Ratana | Nam Pong | Mekong | 1st | Thailand | 5149 | 12117 | NA |  | Multipurpose | 92.30 | 2263 | 0.777 |
| 38. | Lamtakhong | Mun | Mekong | 1st | Thailand | 5156 | 1331 | NA |  | Irrigation | 14.50 | 310 | 0.678 |
| 39. | Lamnangrong | Mun | Mekong | 1st | Thailand | 5162 | 457 | NA |  | Irrigation | 2.78 | 150 | 1.711 |
| 40. | Lam_Pao | Chi | Mekong | 1st | Thailand | 5150 | 6061 | NA |  | Irrigation | 73.62 | 1430 | 0.616 |
| 41. | PakMun | Mun | Mekong | 2nd | Thailand | 5795 | 119794 | 1990 |  | Hydropower | 1524.63 | 229 | 0.005 |
| 42. | Sirindhorn | Lam Dam Noi- Mun Tributary | Mekong | 1st | Thailand | 5796 | 2051 | 1971 |  | Hydropower | 60.25 | 1966 | 1.035 |
| 43. | Lower Sesan II | Sesan | Mekong | 7th | Cambodia | 7303 | 49801 | 2017 |  | Hydropower | 1632.26 | 1790 | 0.035 |
| **Gauging Stations** | | | | | | | | | | | | | |
| 1. | Son Tay | Red Mainstream | Red-Thai Binh | ~100-200 km downstream of Red-Thai Binh cascade | VN |  | 143600 |  |  |  |  |  |  |
| 2. | Ta Pao | La Nga  Tributary | Dong Nai | ~20 km after La Nga cascade | VN |  | 2010 |  |  |  |  |  |  |
| 3. | Voeun Sai | Sesan  Tributary | 2S | ~180 km after Sesan cascade | KH |  | 16300 |  |  |  |  |  |  |
| 4. | Ban Don | Srepok  Tributary | 2S | ~ 22km after Srepok cascade | VN |  | 10700 |  |  |  |  |  |  |
| 5. | Chiang Saen | Mekong Mainstream | Mekong | ~300 km after Lancang cascade | TH |  | 189000 |  |  |  |  |  |  |
| 6. | Nakhom Phanom | Mekong  Mainstream | Mekong | ~150-300km after Nam Ngum, Nam Theun, Nam Mang cascade | TH |  | 373000 |  |  |  |  |  |  |
| 7. | Pakse | Mekong Mainstream | Mekong | ~ 70 km after Mun cascade | LA |  | 545000 |  |  |  |  |  |  |
| 8. | Stung Treng | Mekong Mainstream | Mekong | ~70 km from Lower Sesan 2 | KH |  | 635000 |  |  |  |  |  |  |
| 9. | Kratie | Mekong Mainstream | Mekong | ~300 km downstream of Pakse | KH |  | 646000 |  |  |  |  |  |  |

Note: Year of commission for ungauged reservoirs are retrieved from GRanD database (Lehner et al., 2011). Year retrieved from GRanD is not specified as either year of construction or commission.

# **Table S2.** Performance metrics used in this study.

| **Performance metrics** | **Equations** | **Range** | **Value of perfect agreement** | **References** | **Target variables** |
| --- | --- | --- | --- | --- | --- |
| Correlation coefficient ($CC$) | $\frac{cov (\hat{x},x)}{\hat{\sigma},\sigma}$ | [-1,1] | 1 |  | All |
| Root Mean Square Error $(RMSE)$ | $\sqrt{\frac{\sum_{i=1}^{n} (\hat{x_{i}}-x_{i})^{2}}{n}}$ | [0,$\infty$] | 0 |  | All |
| Normalized Root Mean Square Error ($NRMSE$) | $\frac{RMSE}{x_{max}-x_{min}}$ | [0,1] | 0 |  | All |
| Nash–Sutcliffe efficiency ($NSE$) for normal flows | $1-\frac{\sum_{i=1}^{n} (\hat{x_{i}}-x_{i})^{2}}{(\hat{x_{i}}-{\mu)}^{2}}$ | [$-\infty$,1] | 1 | (Nash and Sutcliffe, 1970) | Streamflow (high flow) |
| $NSE$ for logarithmic transformed flows (${NSE}_{ln}$) | $1-\frac{\sum_{i=1}^{n} (ln(\hat{x_{i}}+\varepsilon)-ln(x_{i}+\varepsilon))^{2}}{ln(\hat{x_{i}}+\varepsilon)-ln(\mu+\varepsilon))^{2}}$ | [$-\infty$,1] | 1 | (Oudin *et al.*, 2006) | Streamflow (low flow) |
| Kling-Gupta Efficiency ($KGE$) | $1 -\sqrt{(CC-1)^{2}+(\alpha-1)^{2}+(\beta-1)^{2}}$ | [$-\infty$,1] | 1 | (Gupta *et al.*, 2009) | Streamflow (overall flow) |
| Relative Error ($RE)$ | $\beta= \frac{\hat{\mu}}{\mu}$  $RE=\left( \beta-1 \right)\times100$ | [$-\infty$,$\infty$] | 0 | (Gupta *et al.*, 2009) | Streamflow |
| Relative Error of Standard Deviation ($RESD$) | $\alpha= \frac{\hat{\sigma}}{\sigma}$; $RESD=\left( \alpha\right)\times100$ | [$-\infty$,$\infty$] | 0 | (Gupta *et al.*, 2009) | Streamflow |
| Note: Variables with the accents $\hat{}$ represent predicted/simulated/remotely sensed data whereas non-accent variables represent observed data. $\sigma$: standard deviation of data; $\mu$: mean metric of data. $\varepsilon$ was set as 0.01 before computing transformations, so evaluating results of zero flows were included and not affected (Nash and Sutcliffe, 1970). | | | | | |

# **Table S3.** Statistical evaluation of Sentinel-1-derived reservoir surface areas, elevations and storage volumes and storage changes. Medians with their standard deviations shown in the parentheses. Unit is provided for median $RMSE$. Mcm stands for million cubic meter.

| **Description** | $\boldsymbol{CC}$ | $\boldsymbol{RMSE}$ | $\boldsymbol{NRMSE}$ |
| --- | --- | --- | --- |
| **ROR (mostly SR < 0.1)** | | | |
| Non-interpolated Areas | 0.3 (0.31) | 1.02 (km^2^) (1.46) | 1.07 (1.07) |
| Interpolated daily surface areas | 0.19 (0.31) | 1.02 (km^2^) (1.45) | 0.99 (1.05) |
| Interpolated daily elevations | 0.19 (0.31) | 2.45 (m) (2.96) | 0.52 (0.77) |
| Interpolated daily storage volumes | 0.19 (0.31) | 7.96 (mcm) (24.98) | 0.57 (0.92) |
| Interpolated daily storage changes | 0.08 (0.012) | 1.94 (mcm) (0.96) | 0.1 (0.02) |
| Interpolated monthly storage changes | 0.38 (0.26) | 0.21 (mcm) (0.18) | 0.27 (0.08) |
| **Within-year or Over-year (SR** $\boldsymbol{\geq}$ **0.1)** | | | |
| Non-interpolated Areas | 0.96 (0.04) | 7.33 (km^2^) (19.24) | 0.21 (0.82) |
| Interpolated daily surface areas | 0.95 (0.04) | 7.85 (km^2^) (19.28) | 0.21 (0.81) |
| Interpolated daily elevations | 0.94 (0.05) | 4.03 (m) (2.83) | 0.19 (0.09) |
| Interpolated daily storage volumes | 0.94 (0.05) | 228.49 (mcm) (301.11) | 0.21 (0.12) |
| Interpolated daily storage changes | 0.44 (0.15) | 17.01 (mcm) (20.93) | 0.07 (0.02) |
| Interpolated monthly storage changes | 0.87 (0.11) | 4.29 (mcm) (5.65) | 0.12 (0.04) |

| **Location** | **Time period** | $\boldsymbol{NSE}$ | $\boldsymbol{NSE}_{\boldsymbol{ln}}$ | $\boldsymbol{KGE}$ | $\boldsymbol{CC}$ | $\boldsymbol{RE}$ | $\boldsymbol{RESD}$ |
| --- | --- | --- | --- | --- | --- | --- | --- |
| Overall Greater Mekong region | Calibration  (2002-2009) | 0.66 (0.29) | NA | 0.76 (0.23) | 0.85 (0.14) | -1.8 (24.28) | -7.31 (21.64) |
|  | Validation  (1991-2001) | 0.65 (0.31) | NA | 0.72 (0.25) | 0.86 (0.11) | 3.05 (28.38) | -6.06 (20.01) |
| **Reservoirs** |  |  |  |  |  |  |  |
| Tuyen Quang-1^st^ reservoir of Gam river | 2016-2018 | 0.7 | 0.69 | 0.67 | 0.85 | -12.04 | -26.58 |
| Thac Ba-1^st^ reservoir of Lo river | 2016-2018 | 0.62 | 0.45 | 0.7 | 0.79 | -4.38 | -20.46 |
| Ban Chat-1^st^ reservoir of Nam Mu river | 2016-2018 | 0.59 | 0.7 | 0.57 | 0.79 | -1.95 | -37.32 |
| Ban Ve-1^st^ reservoir of Ca river | 2016-2018 | 0.64 | 0.79 | 0.56 | 0.84 | -2.09 | -40.31 |
| Ham Thuan-1^st^ reservoir of La Nga river | 2016-2018 | 0.69 | 0.75 | 0.67 | 0.85 | -15.87 | -25.03 |
| Pleikrong-1^st^ reservoir of Sesan river | 2016-2018 | 0.54 | 0.53 | 0.68 | 0.74 | 0.03 | -18.69 |
| Buon Tua Srah-1^st^ reservoir of Srepok river | 2016-2018 | 0.59 | 0.73 | 0.73 | 0.78 | -10.84 | -12.88 |

# **Table S4.** Statistical evaluation of simulated inflows of the first order reservoirs in each cascade system. Medians with their standard deviations shown in the parentheses.

| **Description** | **Time step** | $\boldsymbol{CC}$ | $\boldsymbol{RMSE}$ **(mcm)** | $\boldsymbol{NRMSE}$ |
| --- | --- | --- | --- | --- |
| ROR  (mostly SR < 0.1) | DD | 0.02 (0.03) | 2.02 (1.16) | 0.1 (0.02) |
|  | MO | 0.1 (0.15) | 0.24 (0.42) | 0.38 (0.12) |
| Gauged  Within-year or Over-year  (SR $\geq$ 0.1) | DD | 0.41 (0.19) | 14.24 (24.72) | 0.07 (0.02) |
|  | MO | 0.65 (0.24) | 5.29 (10.59) | 0.19 (0.07) |
| Ungauged  Within-year or Over-year  (SR $\geq$ 0.1) | DD | 0.27 (0.17) | 3.5 (34.19) | 0.14 (0.05) |
|  | MO | 0.57 (0.24) | 1.82 (14.54) | 0.19 (0.07) |
| Note: DD: daily. MO: monthly. At ungauged reservoirs, IROS based $\Delta S$ were compared with remote sensing imagery based $\Delta S$. | | | | |

# **Table S5.** Statistical evaluation of IROS-based $\Delta S$ for both gauged and ungauged reservoirs. Medians with their standard deviations shown in the parentheses. Unit is provided for median $RMSE$. Mcm for million cubic meter.

# **Table S6.** Statistical evaluation of MB-based reservoir outflows. Medians with their standard deviations shown in the parentheses. Unit is provided for median $RMSE$.

| **Description** | **Time step** | $\boldsymbol{NSE}$ | $\boldsymbol{NSE}_{\boldsymbol{ln}}$ | $\boldsymbol{KGE}$ | $\boldsymbol{CC}$ | $\boldsymbol{RE (\%)}$ | $\boldsymbol{RESD (\%)}$ | **RMSE (m^3^/s)** | | $\boldsymbol{NRSME}$ |
| --- | --- | --- | --- | --- | --- | --- | --- | --- | --- | --- |
| **ROR (mostly**  **SR < 0.1)** | DD | 0.46 (0.2) | 0.35 (0.24) | 0.73 (0.13) | 0.76 (0.13) | 2.85 (10) | 0.06 (13.27) | 120.93 (56.28) | | 0.08 (0.06) |
|  | MO | 0.57 (0.16) | 0.7 (0.13) | 0.77 (0.1) | 0.82 (0.08) | 3.28 (10.43) | 1.08 (19.03) | 84.2 (33.24) | | 0.17 (0.05) |
| **Within-year or Over-year**  **(SR** $\boldsymbol{\geq}$ **0.1)** | DD | 0.28 (0.49) | -0.02 (0.17) | 0.55 (0.17) | 0.63 (0.16) | 3.07 (13.91) | -7.44 (19.66) | 192.8 (297.57) | | 0.09 (0.03) |
|  | MO | 0.66 (0.39) | 0.41 (0.3) | 0.74 (0.16) | 0.83 (0.12) | 3.87 (16.17) | -6.16 (17.53) | 105.06 (151.84) | | 0.13 (0.06) |
| Hoa Binh - Cascade Da | DD | 0.45 | 0.23 | 0.64 | 0.68 | 0.01 | -17.01 | 1060.25 | | 0.09 |
|  | MO | 0.78 | 0.70 | 0.88 | 0.89 | 3.63 | -2.30 | 495.441 | | 0.12 |
| Khe Bo - Cascade Ca | DD | 0.72 | 0.48 | 0.76 | 0.86 | 18.29 | -7.93 | 206.489 | | 0.05 |
|  | MO | 0.87 | 0.73 | 0.79 | 0.95 | 19.03 | -7.51 | 111.113 | | 0.07 |
| Da Mi – Cascade La Nga | DD | 0.11 | -0.09 | 0.44 | 0.50 | -11.67 | -20.25 | 29.699 | | 0.21 |
|  | MO | 0.44 | 0.48 | 0.55 | 0.72 | -12.20 | -33.70 | 17.98 | | 0.20 |
| Sesan 4A – Cascade Sesan | DD | 0.39 | -0.18 | 0.68 | 0.69 | 7.35 | -3.18 | 254.3 | | 0.07 |
|  | MO | 0.68 | 0.58 | 0.79 | 0.83 | 8.31 | -9.47 | 149.84 | | 0.10 |
| Srepok 4 – Cascade Srepok | DD | 0.49 | 0.37 | 0.75 | 0.77 | 3.27 | 8.67 | 120.532 | | 0.08 |
|  | MO | 0.56 | 0.72 | 0.77 | 0.82 | 3.29 | 14.91 | 92.208 | | 0.17 |
| Chiang Saen Station – Cascade LanCang | DD | -0.235 | -0.396 | 0.481 | 0.576 | 13.845 | 26.619 | 1422.298 | | 0.206 |
|  | MO | -0.283 | -0.995 | 0.459 | 0.597 | 10.548 | 34.633 | 1128.896 | | 0.286 |
| Note: DD: daily. MO: monthly. | | | | | | | | |  |  |

# **Table S7.** Statistical evaluation of IROS-based reservoir outflows. Medians with their standard deviations shown in the parentheses. Unit is provided for median $RMSE$.

| **Description** | **Time step** | $\boldsymbol{NSE}$ | $\boldsymbol{NSE}_{\boldsymbol{ln}}$ | $\boldsymbol{KGE}$ | $\boldsymbol{CC}$ | $\boldsymbol{RE (\%)}$ | $\boldsymbol{RESD (\%)}$ | **RMSE (m^3^/s)** | $\boldsymbol{NRMSE}$ |  |
| --- | --- | --- | --- | --- | --- | --- | --- | --- | --- | --- |
| **ROR**  **(mostly**  **SR < 0.1)** | DD | 0.51 (0.22) | 0.35 (0.27) | 0.75 (0.2) | 0.77 (0.15) | 1.78 (10.77) | -7.44 (21.2) | 112.57 (47.56) | 0.07 (0.06) |  |
|  | MO | 0.6 (0.21) | 0.72 (0.25) | 0.79 (0.16) | 0.83 (0.11) | 1.78 (10.92) | -1.72 (21.05) | 80.16 (27.42) | 0.16 (0.05) |  |
| **Within-year or Over-year**  **(SR** $\boldsymbol{\geq}$ **0.1)** | DD | 0.28 (0.22) | 0.04 (0.15) | 0.51 (0.14) | 0.59 (0.16) | -1.63 (8.37) | -34.59 (16.1) | 144.83 (302.24) | 0.09 (0.03) |  |
|  | MO | 0.49 (0.23) | 0.36 (0.35) | 0.65 (0.12) | 0.74 (0.13) | -1.56 (8.14) | -21.97 (17.67) | 96.16 (191.61) | 0.14 (0.06) |  |
| Hoa Binh - Cascade Da | DD | 0.38 | 0.38 | 0.49 | 0.62 | -4.08 | -33.01 | 1022.715 | 0.08 |  |
|  | MO | 0.55 | 0.63 | 0.67 | 0.75 | -5.76 | -21.06 | 677.103 | 0.15 |  |
| Khe Bo - Cascade Ca | DD | 0.78 | 0.62 | 0.77 | 0.89 | 15.31 | -13.52 | 179.746 | 0.05 |  |
|  | MO | 0.89 | 0.76 | 0.82 | 0.96 | 15.62 | -7.13 | 101.397 | 0.06 |  |
| Da Mi – Cascade La Nga | DD | 0.10 | 0.02 | 0.30 | 0.45 | -15.84 | -39.21 | 30.334 | 0.21 |  |
|  | MO | 0.25 | 0.26 | 0.44 | 0.61 | -15.73 | -36.73 | 20.778 | 0.23 |  |
| Sesan 4A – Cascade Sesan | DD | 0.61 | 0.28 | 0.63 | 0.79 | 7.16 | -29.65 | 191.075 | 0.05 |  |
|  | MO | 0.72 | 0.67 | 0.67 | 0.87 | 7.18 | -29.26 | 139.618 | 0.10 |  |
| Srepok 4 – Cascade Srepok | DD | 0.56 | 0.38 | 0.78 | 0.78 | 2.25 | -1.10 | 112.003 | 0.08 |  |
|  | MO | 0.64 | 0.77 | 0.82 | 0.83 | 2.31 | 5.38 | 83.25 | 0.16 |  |
| Chiang Saen Station – Cascade LanCang | DD | 0.61 | 0.493 | 0.801 | 0.803 | -1.704 | -1.54 | 738.507 | 0.107 |  |
|  | MO | 0.704 | 0.603 | 0.845 | 0.866 | -1.739 | 7.525 | 551.756 | 0.134 |  |
| **Vietnamese stations** | DD | 0.33 (0.26) | 0.52 (0.42) | 0.64 (0.25) | 0.78 (0.09) | 22.05 (23.18) | -3.96 (22.67) | 120.37 (602.83) | 0.09 (0.02) |  |
|  | MO | 0.36 (0.28) | 0.67 (0.38) | 0.61 (0.23) | 0.84 (0.06) | 21.99 (23.21) | 3.29 (22.59) | 97.22 (429.75) | 0.2 (0.04) |  |
| **International stations** | DD | 0.91 (0.14) | 0.86 (0.17) | 0.83 (0.05) | 0.98 (0.08) | 8.05 (10.59) | -1.54 (7.45) | 2239.53 (1144.44) | 0.07 (0.02) |  |
|  | MO | 0.93 (0.1) | 0.86 (0.12) | 0.83 (0.09) | 0.98 (0.05) | 3.16 (14.89) | 0.46 (14.11) | 1785.27 (1128.23) | 0.07 (0.03) |  |
| Note: DD: daily. MO: monthly. International stations are downstream of ungauged reservoirs. | | | | | | | | | | |


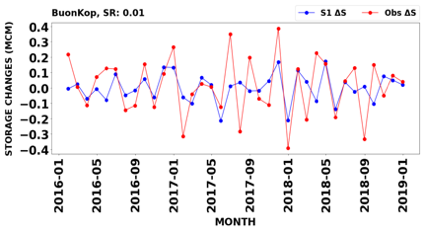

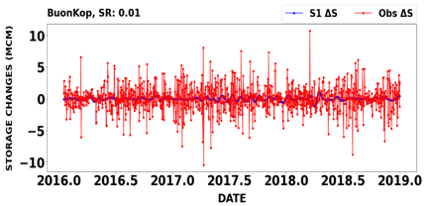

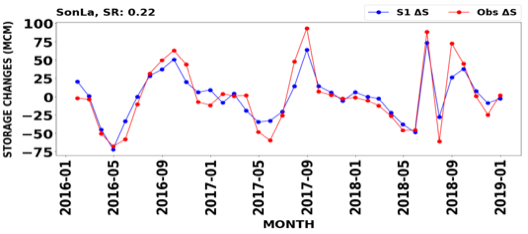

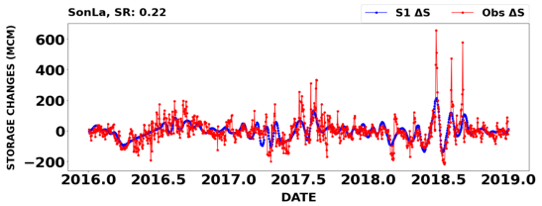

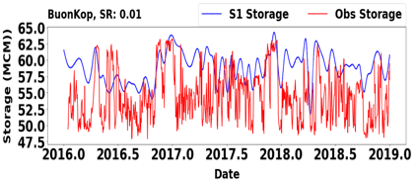

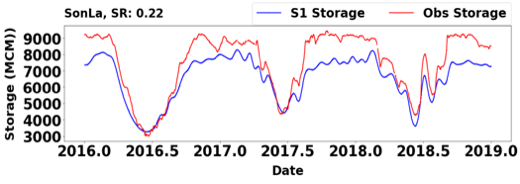

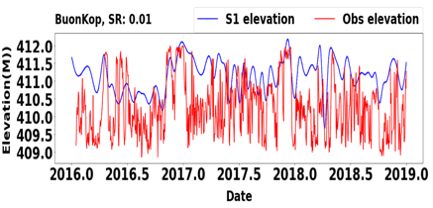

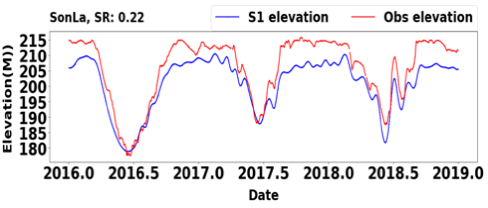

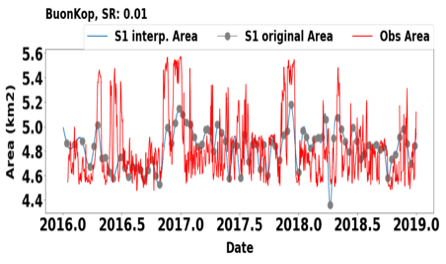

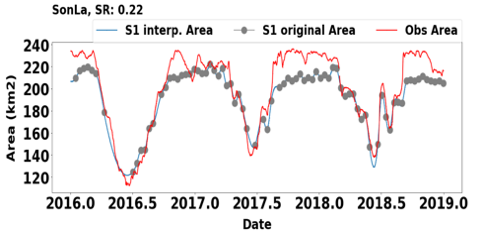

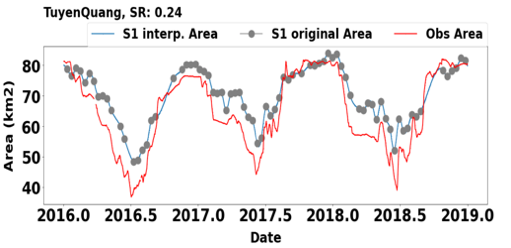

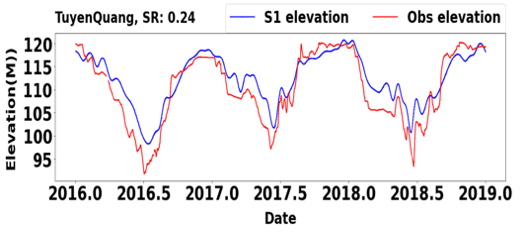

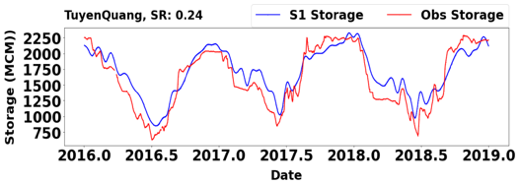

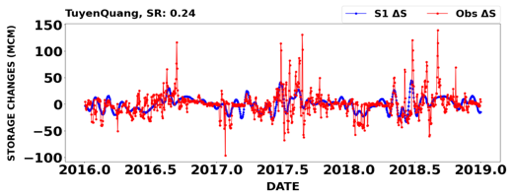

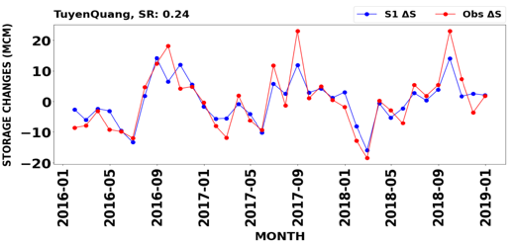


# **Figure S1.** Sentine1-derived time series of the reservoir surface areas, elevations and storage volumes for three sample reservoirs compared with observed (Obs) data. Tuyen Quang is an over-year reservoir, Son La is a within-year reservoir, and Buon Kop is an ROR reservoir. ‘Interp.’ denotes interpolated data. ‘Original’ denotes non-interpolated data. ‘S1’ denotes Sentinel-1. Locations of the reservoirs within the Greater Mekong study region are shown in Figure 1.


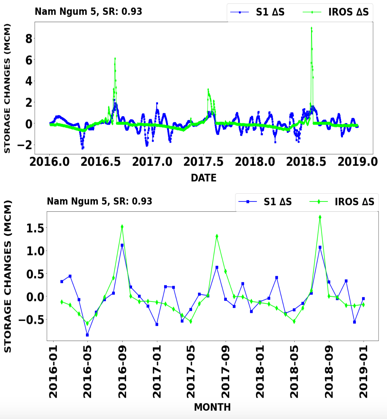

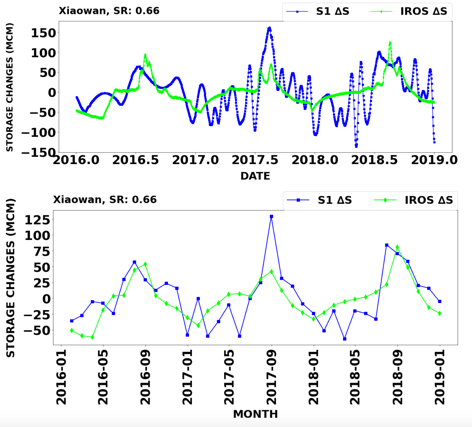

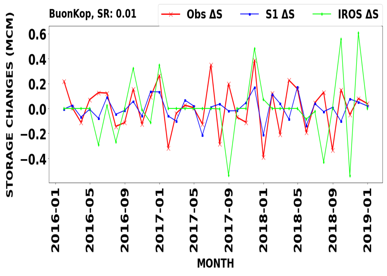

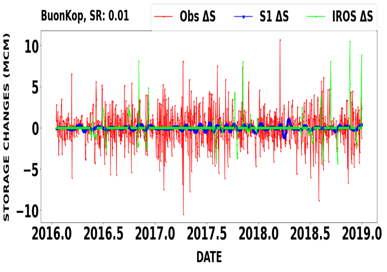

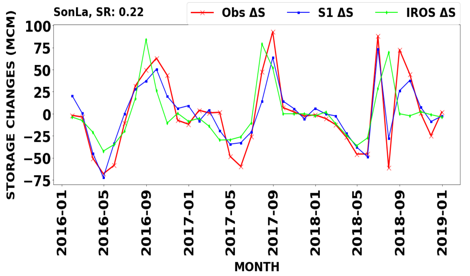

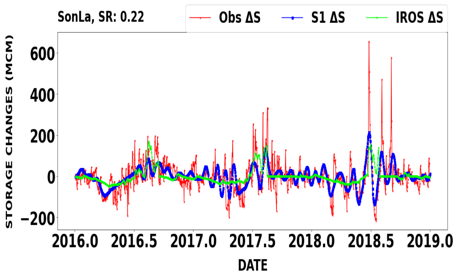

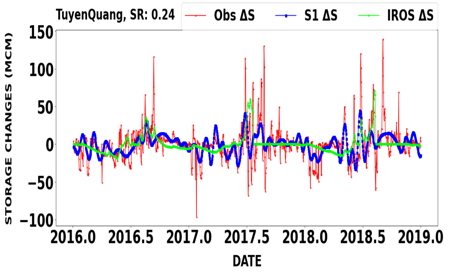

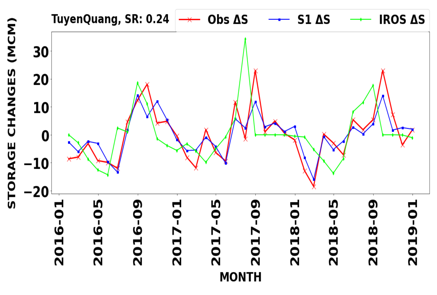

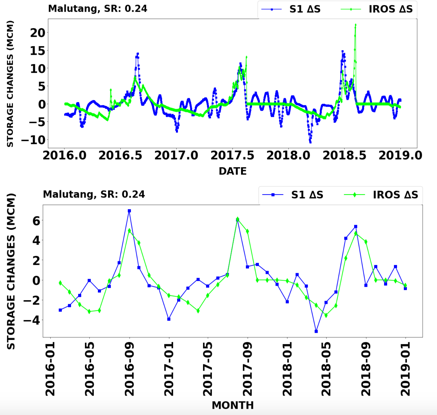


# **Figure S2.** The IROS-based simulated storage changes (IROS $\Delta S$) compared with Sentinel-1-derived (S1 $\Delta S$) and observed (Obs $\Delta S$) storage changes.

**References**

Gupta, H.V., Kling, H., Yilmaz, K.K. and Martinez, G.F. 2009. Decomposition of the mean squared error and NSE performance criteria: Implications for improving hydrological modelling. *Journal of Hydrology*, 377 (1), pp.80–91.

Nash, J.E. and Sutcliffe, J.V. 1970. River flow forecasting through conceptual models part I — A discussion of principles. *Journal of Hydrology*, 10 (3), pp.282–290.

Oudin, L., Andréassian, V., Mathevet, T., Perrin, C. and Michel, C. 2006. Dynamic averaging of rainfall-runoff model simulations from complementary model parameterizations. *Water Resources Research*, 42 (7). [Online]. Available from: https://agupubs.onlinelibrary.wiley.com/doi/abs/10.1029/2005WR004636 [Accessed 2 June 2021].
